# Geranylgeranyl pyrophosphate depletion by statins compromises skeletal muscle insulin sensitivity

**DOI:** 10.1002/jcsm.13061

**Published:** 2022-08-12

**Authors:** Lai Wang, Zuguo Zheng, Lijun Zhu, Lingchang Meng, Hanling Liu, Keke Wang, Jun Chen, Ping Li, Hua Yang

**Affiliations:** ^1^ State Key Laboratory of Natural Medicines, School of Traditional Chinese Pharmacy China Pharmaceutical University Nanjing China

**Keywords:** Statin, GGPP, Skeletal muscle, Insulin resistance, RhoA, RAB8A

## Abstract

**Background:**

Statins are widely prescribed cholesterol‐lowering drugs but have been shown to increase the risk of type 2 diabetes mellitus. However, the molecular mechanisms underlying the diabetogenic effect of statins are still not fully understood.

**Methods:**

The effects of geranylgeranyl transferase I and II (GGTase I and II) inhibition on insulin‐stimulated glucose uptake and GLUT4 translocation, and the dependence of these effects on insulin signalling were investigated in skeletal muscle cells. The protective effects of geranylgeranyl pyrophosphate (GGPP) and its precursor geranylgeraniol (GGOH) on simvastatin‐induced insulin resistance were evaluated *in vitro* and *in vivo*. The effect of GGTase II inhibition in skeletal muscle on insulin sensitivity *in vivo* was confirmed by adeno‐associated virus serotype 9 (AAV9)‐mediated knockdown of the specific subunit of GGTase II, RABGGTA. The regulatory mechanisms of GGTase I on insulin signalling and GGTase II on insulin‐stimulated GLUT4 translocation were investigated by knockdown of RhoA, TAZ, IRS1, geranylgeranylation site mutation of RhoA, RAB8A, and RAB13.

**Results:**

Both inhibition of GGTase I and II mimicked simvastatin‐induced insulin resistance in skeletal muscle cells. GGPP and GGOH were able to prevent simvastatin‐induced skeletal muscle insulin resistance *in vitro* and *in vivo*. GGTase I inhibition suppressed the phosphorylation of AKT (Ser473) (−51.3%, *P* < 0.01), while GGTase II inhibition had no effect on it. AAV9‐mediated knockdown of RABGGTA in skeletal muscle impaired glucose disposal without disrupting insulin signalling *in vivo* (−46.2% for gastrocnemius glucose uptake, *P* < 0.001; −52.5% for tibialis anterior glucose uptake, *P* < 0.001; −17.8% for soleus glucose uptake, *P* < 0.05; −31.4% for extensor digitorum longus glucose uptake, *P* < 0.01). Inhibition of RhoA, TAZ, IRS1, or geranylgeranylation deficiency of RhoA attenuated the beneficial effect of GGPP on insulin signalling in skeletal muscle cells. Geranylgeranylation deficiency of RAB8A inhibited insulin‐stimulated GLUT4 translocation and concomitant glucose uptake in skeletal muscle cells (−42.8% for GLUT4 translocation, *P* < 0.01; −50.6% for glucose uptake, *P* < 0.001).

**Conclusions:**

Geranylgeranyl pyrophosphate regulates glucose uptake via GGTase I‐mediated insulin signalling‐dependent way and GGTase II‐mediated insulin signalling‐independent way in skeletal muscle. Supplementation of GGPP/GGOH could be a potential therapeutic strategy for statin‐induced insulin resistance.

## Introduction

Statins are the most common clinically used lipid‐lowering drug worldwide. Despite their beneficial effects on preventing primary and secondary cardiovascular disease (CVD), emerging data show that statin use has been associated with increased risk of new‐onset type 2 diabetes mellitus (TD2M).^1^ Proposed mechanisms underlying the diabetogenic effect of statins include increased hepatic gluconeogenesis,^2^ NLRP3 inflammasome‐mediated adipose insulin resistance,^3^ skeletal muscle insulin resistance,^4^ reduced β‐cell functional mass,^5^ inhibited browning of adipose tissue,^6^ decreased butyrate production by the gut microbiota,^7^ delayed glucose clearance rate,^8^ and even DNA methylation,^9^ leading to concern about the use of statins in clinical practice. Skeletal muscle is the largest glucose‐consuming and insulin‐sensitive tissue in the human body, where more than 2/3 of the postprandial glucose is transported.^10^ Therefore, skeletal muscle is critical for systematic glucose homeostasis. However, the mechanism underlying statin‐induced insulin resistance in skeletal muscle remains not be fully understood.

Statin‐targeted mevalonate pathway not only controls *de novo* synthesis of cholesterol but also production of an intermediate geranylgeranyl pyrophosphate (GGPP), which is responsible for the geranylgeranylation of specific substrate proteins, including small monomeric GTPases.^11^ Geranylgeranyl diphosphate synthase (GGPS1) is the key enzyme for GGPP biosynthesis.^12^ GGTase II (RAB geranylgeranyl transferase, RABGGTase) specifically catalyses the covalent attachment of GGPP to the cysteine of a carboxyl‐terminal CC or CXC motif of RAB proteins, which belong to the Ras superfamily of small monomeric GTPases and extensively involve in intracellular membrane trafficking of membrane‐bound organelles and vesicles,^13^ while GGTase I is broadly responsible for catalysing the covalent attachment of GGPP to the cysteine of a C‐terminal CAAX motif of other small GTPases.^14^ GGPP depletion underpins statins‐induced IL‐1β‐dependent adipocyte insulin resistance. β‐Cell‐specific ablation of GGPS1 results in β‐cell dysfunction mediated by impairing RAB27A geranylgeranylation.^15^ Atorvastatin dysregulates mammalian target of rapamycin (mTOR) signalling and reduces β‐cell functional mass by inhibiting the geranylgeranylation of RAB5A.^5^ These reports suggest that GGPP regulates insulin activity in adipocyte and β‐cell, but the role of GGPP in insulin activity in skeletal muscle is still unclear.

Insulin functions via binding its receptors to activate the receptor tyrosine kinase, leading to the tyrosine phosphorylation of insulin receptor substrates (IRSs), which then initiate phosphatidylinositol 3‐kinase (PI3K)‐AKT signalling cascade to regulate muscle glucose homeostasis.^16^ It has been reported that statins regulate insulin sensitivity by TAZ‐mediated IRS1 expression in skeletal muscle.^4^ Abundant evidences have suggested that TAZ is a downstream target of RhoA, a member of Rho family small GTPases that belong to the Ras superfamily of small monomeric GTPases.^17^ In addition, activated AKT phosphorylates and inactivates AS160, a GTPase‐activating protein for RAB proteins, which enables RAB proteins exchange from an inactive GDP binding form to an active GTP binding form.^18^ Up to now, several candidate RAB proteins including RAB3,^19^ RAB4,^20^ RAB8A,^21^ RAB10,^22^ RAB13,^21^ and RAB14^23^ have been identified to participate in insulin‐stimulated GLUT4 storage vesicles (GSVs) mobilization in adipocytes and skeletal muscle cells, among which RAB8A and RAB13 are predominant in skeletal muscle.^21^ Thus, it is possible that statins might induce insulin resistance in skeletal muscle by disturbing the geranylgeranylation of small GTPases, but this hypothesis has not yet been tested.

In this study, both *in vivo* and *in vitro* approaches were employed to test this hypothesis. We herein show that statin‐targeted mevalonate pathway regulates insulin sensitivity of skeletal muscle via RhoA geranylgeranylation‐mediated insulin signalling‐dependent manner and RAB8A geranylgeranylation‐mediated insulin signalling‐independent manner.

## Methods

For detailed methods, please refer to the supporting information.

### Animal experiments

Male C57BL/6J mice (8 weeks, 20 ± 2 g) used in all animal experiments were obtained from Laboratory Animal Center of Yangzhou University (Yangzhou, China). The animal studies were approved by the Animal Ethics Committee of China Pharmaceutical University. All mice were kept in an air‐conditioned animal quarter at a temperature of 25 ± 2°C and a relative humidity of 50 ± 10% with 12 h light/dark cycles for 1 week before experiments and allowed water and standard chow ad libitum.

### Glucose tolerance and insulin tolerance tests

Glucose tolerance and insulin tolerance tests were performed as previously described.^4^

### Cell culture

The mouse myoblast C2C12 cell line was purchased from American Type Culture Collection (ATCC, USA). Cell culture and differentiation were performed as described earlier.^4^

### 2‐NBDG uptake assay

2‐NBDG uptake assay was performed as described earlier.^24^

### *In vivo* 2‐DG uptake assay

Mice were fasted for 16 h, and then 2‐DG (2 g/kg) was intraperitoneally injected. Thirty minutes later, mice were anaesthetized and euthanized. Skeletal muscle including gastrocnemius, tibialis anterior, soleus, and extensor digitorum longus was harvested, and intramuscular 2‐DG content was measured using Glucose Uptake Colorimetric Kit (Biovision, K676‐100) according to the manufacturer's instructions.

### Histological analysis of skeletal muscle

Gastrocnemius muscle was fixed immediately after euthanasia in 4% paraformaldehyde at 4°C overnight and embedded in paraffin wax (Sigma‐Aldrich, 327204). Paraffin sections (5 μm) were cut and mounted on glass slides for haematoxylin and eosin (H&E) staining.

### Real‐time quantitative polymerase chain reaction

Real‐time quantitative polymerase chain reaction (RT‐qPCR) was performed as described previously.^25^ The primer sequences used in this study were listed in Supporting information, *Table* *S3*. For detailed methods, please refer to the supporting information.

### Isolation of plasma membrane fractionation

The Plasma Membrane Protein Extraction Kit (ab65400, Abcam) was used to isolate plasma membrane proteins from C2C12 myotubes and primary mouse myotubes according to the manufacturer's instruction.

### Western blot analysis

Western blot was performed as described previously.^25^ For detailed methods, please refer to the supporting information.

### siRNA and plasmid transfection

C2C12 myotubes and primary mouse myotubes were transfected with siRNA duplexes or plasmids using Lipofectamine 3000 (Invitrogen, Carlsbad, CA, USA) as described earlier.^25^ For transfection of siRNA, 5 nmol of siRNA was used for per well of six‐well cell culture plate. For transfection of plasmids, 1 μg of plasmid DNA was used for per well of six‐well cell culture plate.

### Construction of C2C12 myoblast expressing eGFP–GLUT4

The eGFP–GLUT4 lentivirus was purchased from Shanghai GenePharma Co., Ltd. The cells were seeded at a density of 5 × 10^5^ per well in a small culture dish 24 h before transfection to achieve more than 30% confluence. Twenty microlitres of eGFP–GLUT4 lentivirus and 20 μL of scrambled sequence lentivirus were added into 4 mL of fresh medium individually, and then added 4 μL of polybrene (Santa Cruz Biotechnology, Santa Cruz, CA, USA) after 24 h of treatment, lentivirus medium was replaced by fresh medium.

### Triton X‐114 partition

Unprocessed RhoA, RAB8A, and RAB13 and geranylgeranylated RhoA, RAB8A, and RAB13 were separated by the Triton X‐114 partition method as described previously with appropriate modification.^26^ For detailed methods, please refer to the supporting information.

### Statistical analysis

Data were analysed using the GraphPad Prism 8 software (San Diego, CA, USA). All data were expressed as the means ± standard error of the mean (SEM). Student's *t*‐test and one‐way ANOVA were used to calculate statistical significance. A value of *P* < 0.05 meant significant; values of *P* < 0.01, *P* < 0.001, and *P* < 0.0001 meant highly significant; ns meant no significance.

## Results

### Geranylgeranyl pyrophosphate depletion by statins impaired glucose disposal in skeletal muscle cells *in vitro* and *in vivo*

In our previous study addressing statin‐induced skeletal muscle atrophy,^25^ it was unexpected to observe that administration of simvastatin (80 mg/kg/day) for 8 weeks led to increased fasting blood glucose and impaired glucose tolerance and insulin tolerance in C57BL/6J mice fed with normal diet (*Figure* S1A–S1C). Likewise, administration of lovastatin (125 mg/kg/day) for 8 weeks resulted in increased fasting blood glucose and impaired glucose tolerance and insulin tolerance in high‐fat diet‐induced obese mice (*Figure* S1D–S1F). These *in vivo* experiments imply that statins possess diabetogenic effect. Then the effect of statins on insulin‐stimulated glucose uptake was investigated in C2C12 myotubes and primary mouse myotubes. Based on lipophilicity, eight tested statins could be divided into lipophilic statins and hydrophilic statins.^25^ Almost all statins inhibited insulin‐stimulated glucose uptake except pravastatin (*Figure* S1G). Simvastatin was selected to address our following experiments on account of its strongest lipophilicity among tested statins, and it inhibited insulin‐stimulated glucose uptake in C2C12 myotubes in a dosage‐dependent manner (*Figure* S1H). The aforementioned experiments demonstrate that statins possess diabetogenic effect *in vivo* and could inhibit insulin‐stimulated glucose uptake in skeletal muscle cells *in vitro*.

**Figure 1:**
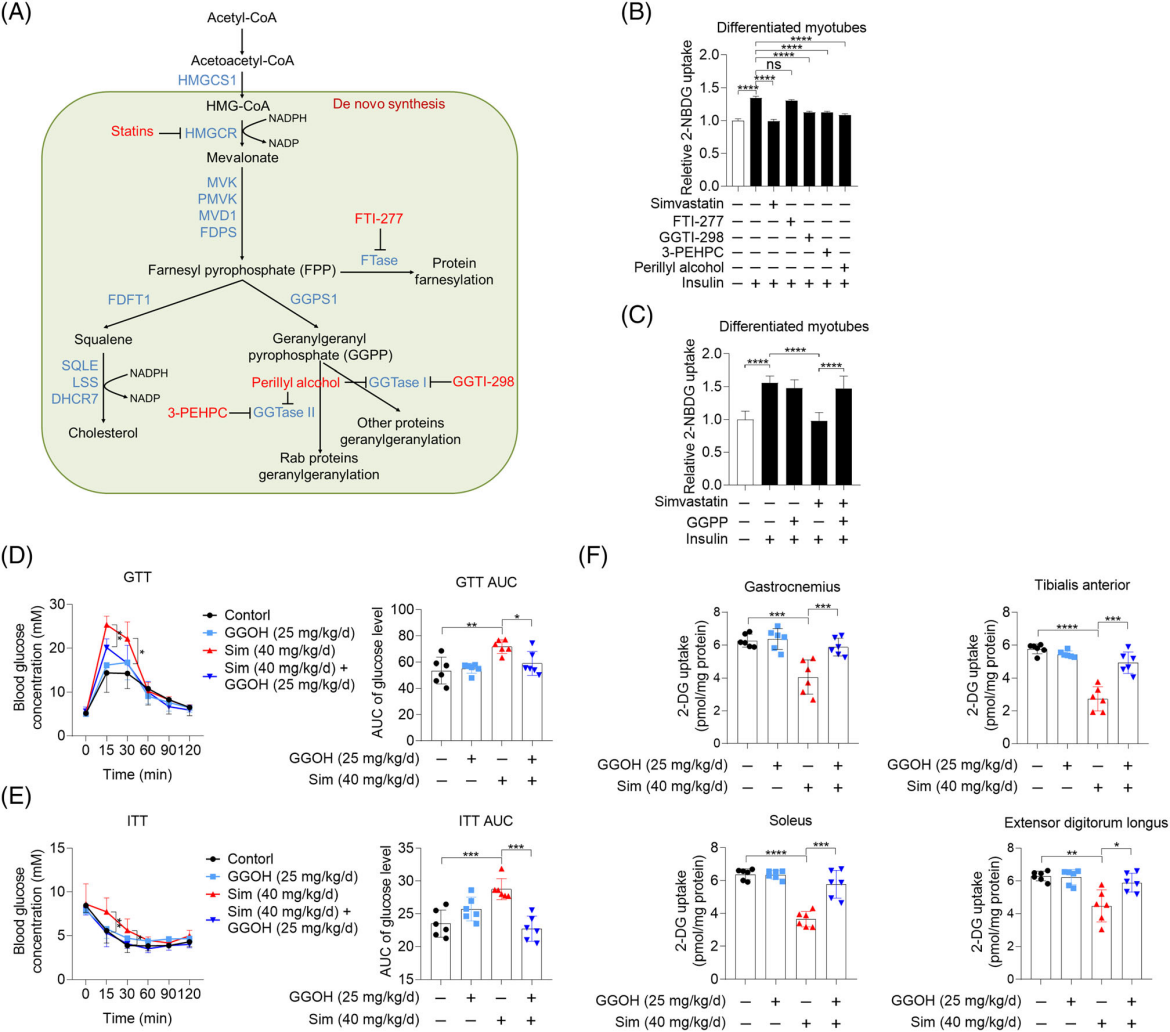
Geranylgeranyl pyrophosphate (GGPP) depletion by statins inhibits glucose uptake in skeletal muscle cells *in vitro* and *in vivo*. *(A)* Mevalonate pathway. *(B)* C2C12 myotubes were pretreated with 10 μM simvastatin, 10 μM FTI‐277, 10 μM GGTI‐298, 1.5 mM 3‐PEHPC, and 1 mM perillyl alcohol for 24 h, then cells were exposed to 2‐NBDG containing 100 nM insulin for 30 min, and 2‐NBDG uptake was measured by fluorescence detection (*n* = 3). *(C)* C2C12 myotubes were treated with 10 μM GGPP, 10 μM simvastatin, and 10 μM GGPP combined with 10 μM simvastatin for 24 h, then cells were exposed to 2‐NBDG containing 100 nM insulin for 30 min, and 2‐NBDG uptake was measured by fluorescence detection (*n* = 3). *(D–F)* Male C57BL/6J mice (20 ± 2 g) were randomly grouped (*n* = 6). After administration of geranylgeraniol (GGOH) (25 mg/kg/day), simvastatin (40 mg/kg/day), and GGOH combined with simvastatin for 3 weeks, mice were subjected with experiments below. *(D)* Glucose tolerance test (GTT) and GTT AUC. *(E)* Insulin tolerance test (ITT) and ITT AUC. *(F)* After the measurement of GTT and ITT, mice were fasted for 16 h, and then mice were administrated with 2‐DG (2 g/kg body weight) via intraperitoneal injection. Mice were sacrificed 30 min later after the injection. The uptake of 2‐DG in gastrocnemius, tibialis anterior, soleus, and extensor digitorum longus was measured using Glucose Uptake Colorimetric Assay Kit. Data represented the mean ± SEM. Statistical analysis was performed with one‐way ANOVA. *P < 0.05; **P < 0.01; ***P < 0.001; ****P < 0.0001.

To investigate whether the depletion of farnesyl pyrophosphate (FPP) and GGPP, two by‐products of cholesterol synthesis pathway, was responsible for simvastatin‐caused inhibition of insulin‐stimulated glucose uptake in skeletal muscle cells, farnesyl transferase (FTase) inhibitor (FTI‐277),^27^ GGTase I inhibitor (GGTI‐298),^27^ GGTase II inhibitor (3‐PEHPC),^28^ and perillyl alcohol, an inhibitor of both GGTase I and GGTase II,^27^ were employed (*Figure* 1A). As shown in *Figure* 1B, GGTI‐298, 3‐PEHPC, and perillyl alcohol mimicked the inhibitory effect of simvastatin on insulin‐stimulated glucose uptake in C2C12 myotubes, while FTI‐277 failed to do so. To further determine the regulatory effects of GGTase I and GGTase II on insulin sensitivity in skeletal muscle cells, genetic inhibition of GGTase I and GGTase II using siRNAs targeting the specific subunit of GGTase I, PGGT1B,^29^ and the specific subunit of GGTase II, RABGGTA,^30^ was performed, with siRNA‐mediated knockdown of GGPS1, the key enzyme for GGPP biosynthesis, as a positive control. siGGPS1#3, siPGGT1B#2, and siRABGGTA#2 were picked to address experiments because of the highest knockdown efficiency among three designed siRNAs (*Figure* S2A–S2C). As shown in *Figure* S2D, both knockdown of PGGT1B and RABGGTA diminished insulin‐stimulated glucose uptake in C2C12 myotubes. GGPP supplementation reversed simvastatin and GGPS1 knockdown‐caused inhibition of insulin‐stimulated glucose uptake (*Figures* 1C and S2E). As expected, GGPP failed to reverse GGTI‐298, 3‐PEHPC, perillyl alcohol, PGGT1B knockdown, and RABGGTA knockdown‐caused inhibition of insulin‐stimulated glucose uptake (*Figure* S3A–S3E). In addition, cholesterol was not able to reverse decreased insulin‐stimulated glucose uptake caused by simvastatin in C2C12 myotubes, reinforcing that simvastatin‐caused inhibition of insulin‐stimulated glucose uptake in C2C12 myotubes was a non‐cholesterol‐lowering effect (*Figure* S3F). To further confirm the beneficial effect of GGPP on statin‐induced insulin resistance *in vivo*, geranylgeraniol (GGOH), which could be converted to GGPP, was used.^31^ We observed that both impaired glucose tolerance and insulin tolerance by simvastatin treatment were improved by GGOH (*Figure* 1D and 1E). Decreased postprandial glucose uptake of skeletal muscle was also largely reversed by GGOH administration (*Figure* 1F). Furthermore, despite well‐established statin‐associated muscle symptoms (SAMS), short exposure to simvastatin with low dosage (40 mg/kg/day) for 21 days did not trigger any SAMS, which was reflected by unchanged body weight, muscle mass, and muscle fibre type composition (*Figure* S4A and S4C–S4E). GGOH had no effect on body weight and insulin secretion (*Figure* S4A and S4B), as well as muscle mass (*Figure* S4C and S4D) and muscle fibre type composition (*Figure* S4E). Taken together, these results demonstrate that GGPP plays a key role in insulin‐stimulated glucose uptake.

### Impaired insulin‐stimulated GLUT4 translocation to the plasma membrane caused by simvastatin was restored by geranylgeranyl pyrophosphate

**Figure 2:**
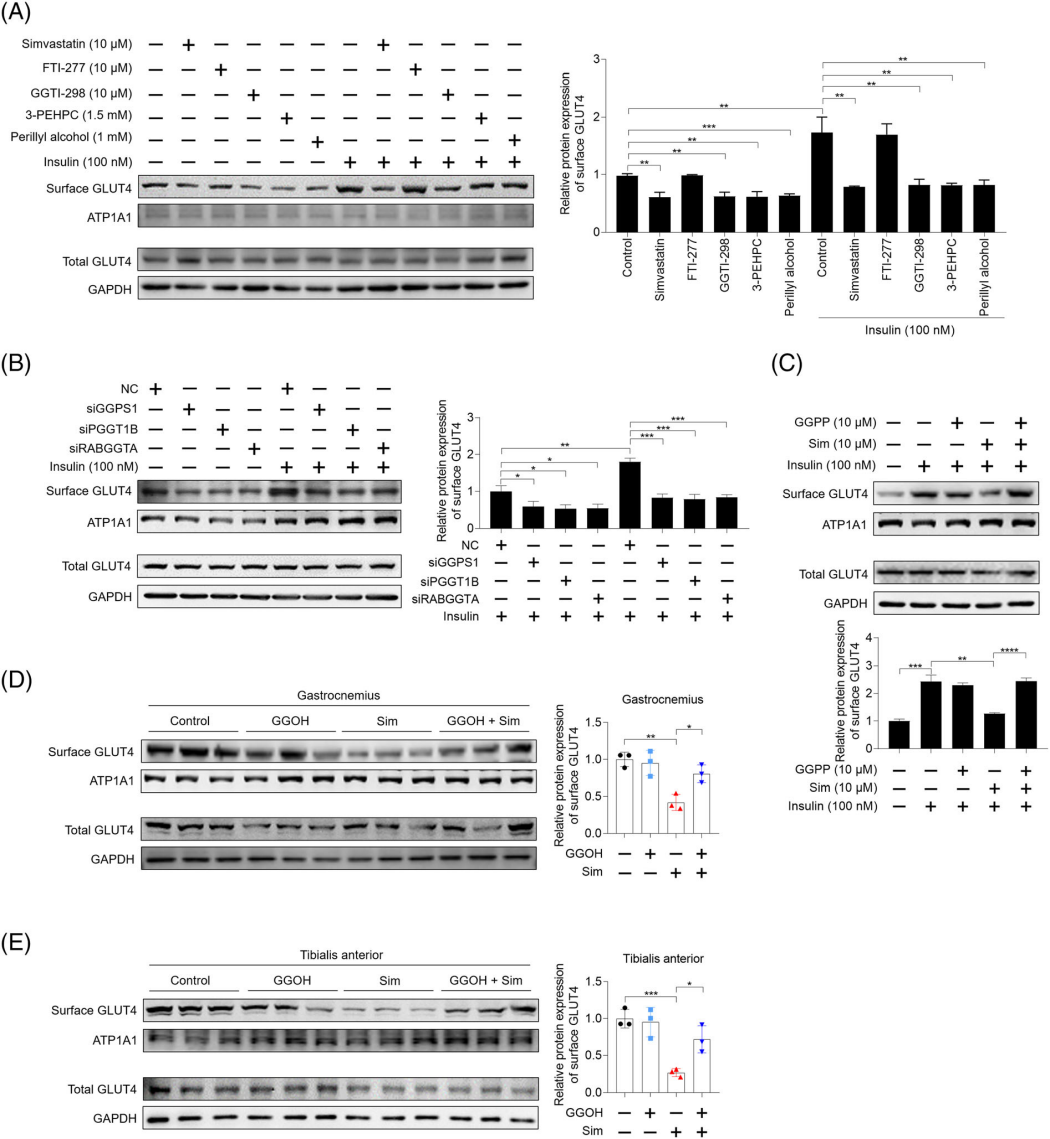
Impaired GLUT4 translocation to plasma membrane caused by simvastatin is restored by geranylgeranyl pyrophosphate (GGPP). *(A)* C2C12 myotubes were pretreated with 10 μM simvastatin, 10 μM FTI‐277, 10 μM GGTI‐298, 1.5 mM 3‐PEHPC, and 1 mM perillyl alcohol for 24 h. Then cells were incubated with or without 100 nM insulin for 30 min, total protein and plasma membrane protein were harvested, and GLUT4 expression was analysed by western blot, with GAPDH as the loading control (*n* = 3). *(B)* C2C12 myotubes were previously transfected with siRNAs targeting GGPS1, PGGT1B, and RABGGTA, respectively, for 48 h. Then cells were incubated with or without 100 nM insulin for 30 min, then total protein and plasma membrane protein were harvested, and GLUT4 expression was analysed by western blot, with GAPDH as the loading control (*n* = 3). *(C)* C2C12 myotubes were pretreated with 10 μM GGPP, 10 μM simvastatin, and 10 μM GGPP combined with 10 μM simvastatin for 24 h, and then cells were treated with 100 nM insulin for 30 min. Total protein samples and plasma membrane protein samples were harvested, and GLUT4 expression was analysed by western blot, with GAPDH as the loading control (*n* = 3). *(D, E)* Male C57BL/6J mice (20 ± 2 g) were randomly grouped (*n* = 6). After administration of geranylgeraniol (GGOH) (25 mg/kg/day), simvastatin (40 mg/kg/day), and GGOH combined with simvastatin for 3 weeks, mice were sacrificed and membrane GLUT4 expression in gastrocnemius *(D)* and tibialis anterior *(E)* was analysed by western blot, with GAPDH as the loading control (*n* = 3). Data represented the mean ± SEM. Statistical analysis was performed with one‐way ANOVA. *P < 0.05; **P < 0.01; ***P < 0.001; ****P < 0.0001; ns denotes no significance.

Given the central role of GLUT4 translocation to the plasma membrane in insulin‐stimulated glucose uptake in skeletal muscle, we next explored whether GGPP involved in insulin‐stimulated GLUT4 mobilization. We observed that GGTI‐298, 3‐PEHPC, and perillyl alcohol markedly decreased surface GLUT4 expression in both basal and insulin‐stimulated conditions without influencing total GLUT4 expression in C2C12 myotubes, while FTI‐277 showed no effect (*Figure* 2A). Decreased surface GLUT4 expression was also visualized in C2C12 myoblasts stably expressing eGFP–GLUT4 (*Figure* S5A). Consistently, genetic inhibition of GGPS1, PGGT1B, and RABGGTA using specific siRNAs also decreased surface GLUT4 expression (*Figures* 2B and S5B). Simvastatin‐triggered inhibition of insulin‐stimulated GLUT4 translocation was restored by GGPP supplementation (*Figure* 2C). Moreover, decreased postprandial surface GLUT4 expression in skeletal muscle after simvastatin treatment was also prevented by GGOH treatment (*Figure* 2D and 2E). Taken together, these data show that GGPP improves insulin‐stimulated glucose uptake via restoring insulin‐stimulated GLUT4 translocation in skeletal muscle cells.

### Insulin signalling is not necessary for simvastatin‐induced insulin resistance in skeletal muscle

**Figure 3:**
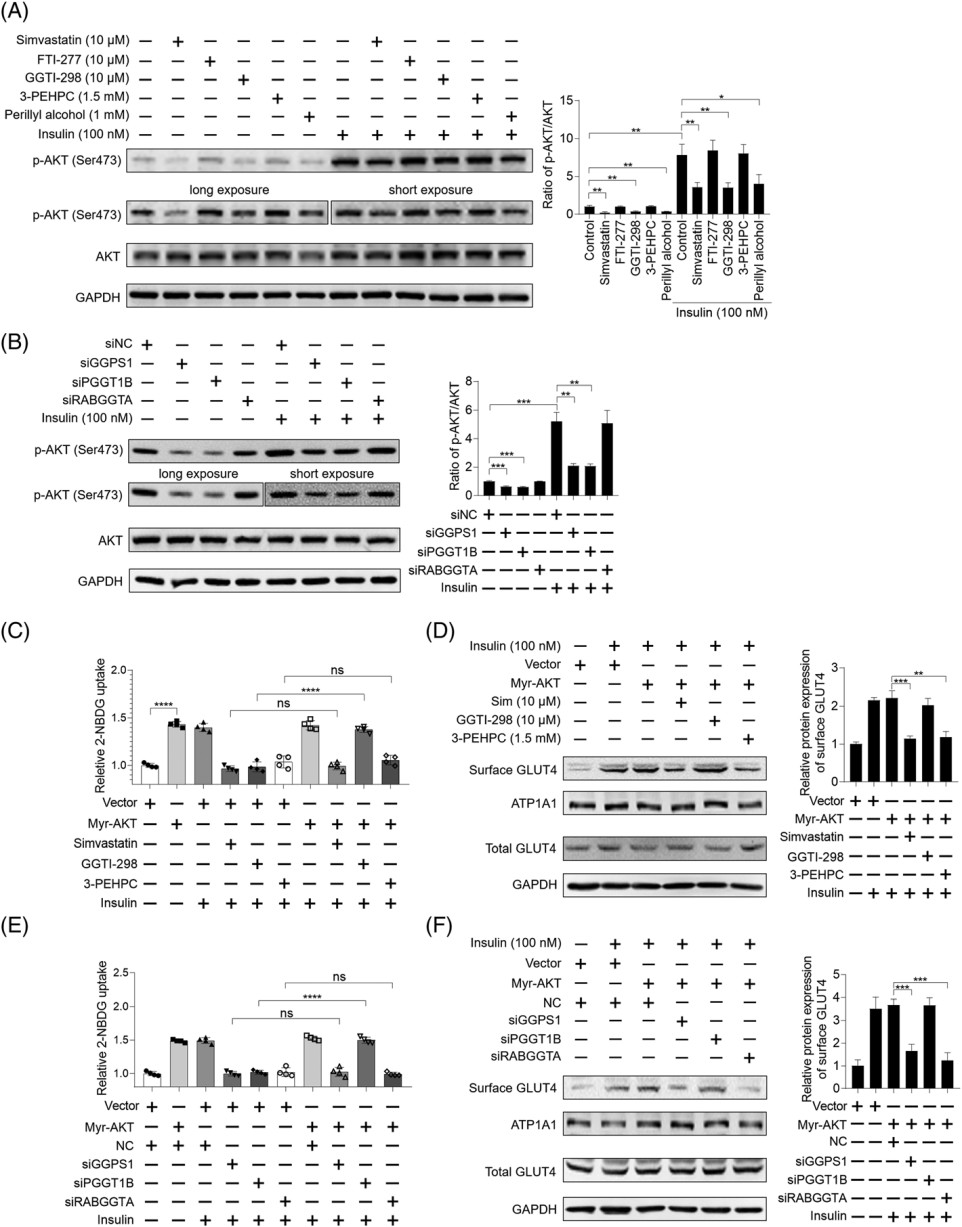
Insulin signalling is not necessary for simvastatin‐caused inhibition of insulin‐stimulated glucose uptake in skeletal muscle cells. *(A)* C2C12 myotubes were pretreated with 10 μM simvastatin, 10 μM FTI‐277, 10 μM GGTI‐298, 1.5 mM 3‐PEHPC, and 1 mM perillyl alcohol for 24 h. Then cells were incubated with or without 100 nM insulin for 30 min, total protein was harvested, and the expression of indicated proteins was analysed by western blot, with GAPDH as the loading control (*n* = 3). Long exposure of p‐AKT (Ser473) for 30 s and short exposure of p‐AKT (Ser473) for 5 s were shown. *(B)* C2C12 myotubes were previously transfected with siRNAs targeting GGPS1, PGGT1B, and RABGGTA, respectively, for 48 h. Then cells were incubated with or without 100 nM insulin for 30 min, total protein was harvested, and the expression of indicated proteins was analysed by western blot, with GAPDH as the loading control (*n* = 3). Long exposure of p‐AKT (Ser473) for 30 s and short exposure of p‐AKT (Ser473) for 5 s were shown. *(C)* C2C12 myotubes were previously transfected with or without 1 μg Myr‐AKT plasmid using Lipofectamine 3000 for 48 h. Then the cells were treated with 10 μM simvastatin, 10 μM FTI‐277, 10 μM GGTI‐298, 1.5 mM 3‐PEHPC, and 1 mM perillyl alcohol for another 24 h. Cells were exposed to 2‐NBDG containing 100 nM insulin for 30 min, and 2‐NBDG uptake was measured by fluorescence detection (*n* = 3). *(D)* C2C12 myotubes were previously transfected with 1 μg Myr‐AKT plasmid using Lipofectamine 3000 for 48 h. Then the cells were treated with 10 μM simvastatin, 10 μM FTI‐277, 10 μM GGTI‐298, 1.5 mM 3‐PEHPC, and 1 mM perillyl alcohol for another 24 h. Cells were incubated with 100 nM insulin for 30 min, then protein samples were harvested, and the expression of indicated proteins was checked by western blot, with GAPDH as the loading control (*n* = 3). *(E)* C2C12 myotubes were previously transfected with or without 1 μg Myr‐AKT plasmid using Lipofectamine 3000 for 48 h. Then cells were transfected with siRNAs targeting GGPS1, PGGT1B, and RABGGTA, respectively, for another 48 h. Cells were exposed to 2‐NBDG containing 100 nM insulin for 30 min, and 2‐NBDG uptake was measured by fluorescence detection (*n* = 3). *(F)* C2C12 myotubes were previously transfected with or without 1 μg Myr‐AKT plasmid using Lipofectamine 3000 for 48 h. Then cells were transfected with siRNAs targeting GGPS1, PGGT1B, and RABGGTA, respectively, for another 48 h. Cells were incubated with 100 nM insulin for 30 min, protein samples were harvested, and the expression of indicated proteins was checked by western blot, with GAPDH as the loading control (*n* = 3). Data represented the mean ± SEM. Statistical analysis was performed with one‐way ANOVA. *P < 0.05; **P < 0.01; ***P < 0.001; ****P < 0.0001; ns denotes no significance.

To investigate whether GGTase I/II inhibition‐caused insulin resistance could be attributed to disrupted insulin signalling, the effect of these inhibitors on the phosphorylation of AKT Ser473 site in skeletal muscle cells was checked. The result showed that GGTI‐298 and perillyl alcohol mimicked the inhibition of simvastatin on the phosphorylation of AKT at both normal and insulin‐stimulated conditions, while 3‐PEHPC had no influence in C2C12 myotubes (*Figure* 3A). In addition, knockdown of GGPS1 and PGGT1B inhibited the phosphorylation of AKT Ser473 site, but RABGGTA knockdown failed in C2C12 myotubes (*Figure* 3B). These findings were also confirmed in primary mouse myotubes (*Figure* S6A and S6B). The aforementioned data imply that GGTase I regulates GLUT4 translocation in an insulin signalling‐dependent manner, while GGTase II regulates GLUT4 translocation in an insulin signalling‐independent manner. In order to reinforce this hypothesis, Myr‐AKT, a consistently activated form of AKT, was employed to consistently activate insulin signalling.^32^ As shown in *Figure* S7A, Myr‐AKT kept high kinase activity and activated insulin signalling, which be characterized by increased phosphorylation of two AKT downstream targets, AS160 and FOXO1. In Myr‐AKT‐expressed C2C12 myotubes, simvastatin could not suppress the phosphorylation of AS160, the key molecular of insulin signalling (*Figure* S7B). In C2C12 myotubes, Myr‐AKT significantly reversed GGTI‐298 and PGGT1B knockdown‐induced decreases of insulin‐stimulated glucose uptake and GLUT4 translocation but showed no effect on simvastatin, 3‐PEHPC, GGPS1 knockdown, and RABGGTA knockdown‐induced decreases of insulin‐stimulated glucose uptake and GLUT4 translocation (*Figures* 3C–3F, S5C, and S5D).

**Figure 4:**
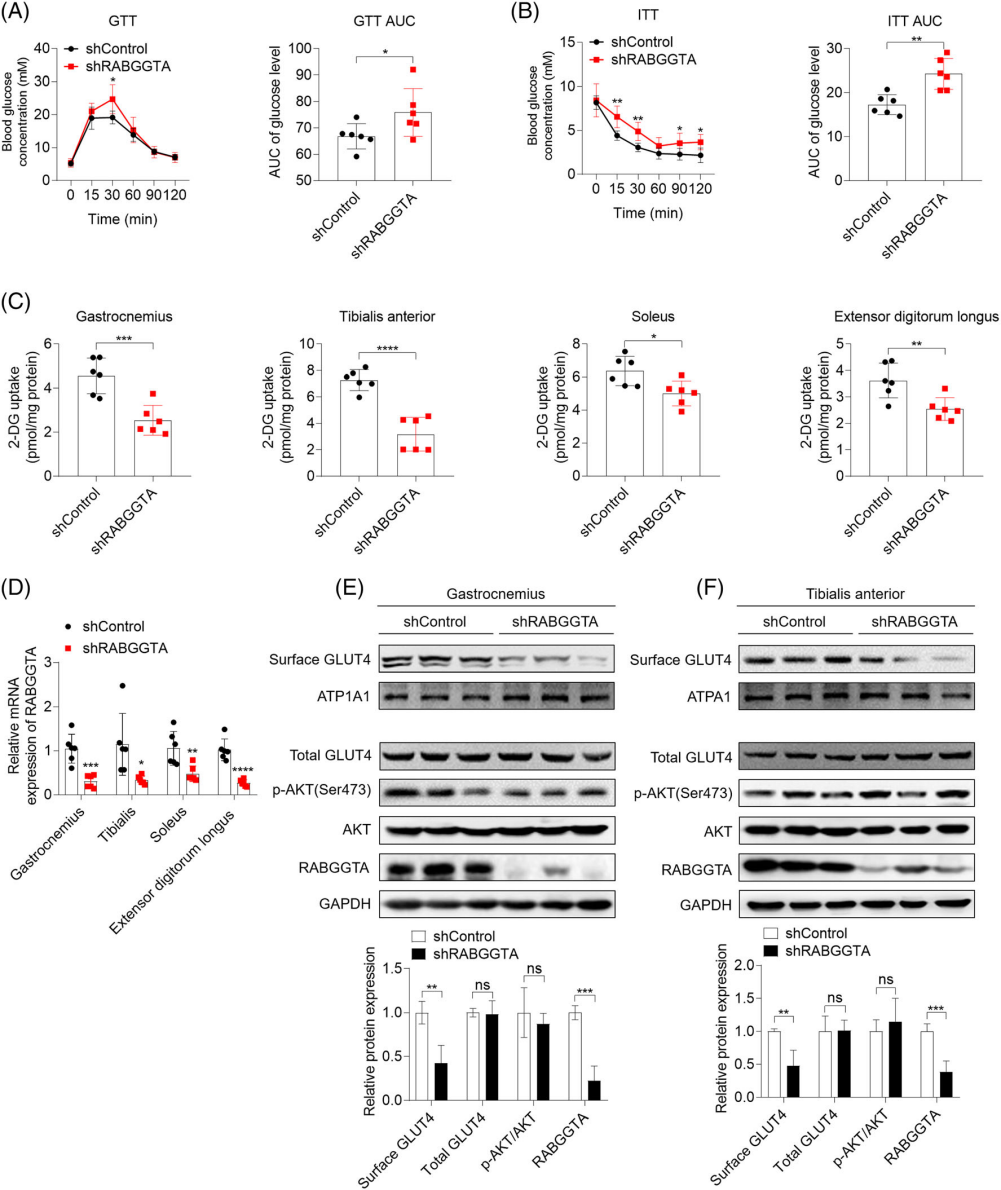
Adeno‐associated virus serotype 9 (AAV9)‐mediated knockdown of RABGGTA in skeletal muscle causes insulin resistance without disturbing insulin signalling *in vivo*. Mice were subjected a week of adjustable feeding and then were divided into two groups including shControl group and shRABGGTA group (*n* = 6). Posterior limbs of mice in shControl group and shRABGGTA group were infected with control AAV9 and shRABGGTA AAV9, respectively, through *in situ* injection. Four weeks after the infection, mice were subjected with experiments as follows. *(A)* Glucose tolerance test (GTT) and GTT AUC. *(B)* Insulin tolerance test (ITT) and ITT AUC. *(C)* Mice were fasted for 16 h before intraperitoneal injection of 2‐DG (2 g/kg body weight). Thirty minutes after the injection, mice were sacrificed and skeletal muscle including gastrocnemius, soleus, tibialis anterior, and extensor digitorum longus was obtained. 2‐DG concentration in each muscle was measured using Glucose Uptake Colorimetric Assay Kit. *(D)* The mRNA expression of RABGGTA in skeletal muscle was quantified by RT‐qPCR. *(E, F)* The expression of indicated proteins in gastrocnemius *(E)* and tibialis anterior *(F)* was detected by western blot, with GAPDH as the loading control of total protein and ATP1A1 as the loading control of plasma membrane protein (*n* = 3). Data represented the mean ± SEM. Statistical analysis was performed with one‐way ANOVA. *P < 0.05; **P < 0.01; ***P < 0.001; ****P < 0.0001; ns denotes no significance.

To further verify the finding of GGTase II‐mediated insulin signalling‐independent way in mevalonate pathway‐regulated insulin sensitivity, adeno‐associated virus serotype 9 (AAV9)‐mediated RABGGTA knockdown was performed via *in situ* skeletal muscle injection of posterior limbs (*Figure* S8A). Four weeks after injection, mice showed impaired glucose tolerance and insulin tolerance (*Figure* 4A and 4B). Consistently, postprandial glucose uptake of skeletal muscle was also decreased (*Figure* 4C). The results of RT‐qPCR and western blot showed that RABGGTA was effectively down‐regulated in skeletal muscle (*Figure* 4D–4F). Consistent with *in vitro* findings, skeletal muscle RABGGTA knockdown suppressed postprandial surface GLUT4 expression without disturbing insulin signalling (*Figure* 4E and 4F). In addition, skeletal muscle RABGGTA knockdown had no effects on body weight, serum insulin levels, muscle masses, and muscle fibre type composition (*Figure* S8B–S8F).

In conclusion, the aforementioned data demonstrate that GGPP regulates insulin sensitivity via GGTase I‐mediated insulin signalling‐dependent way and GGTase II‐mediated insulin signalling‐independent way.

### Geranylgeranyl transferase I‐mediated geranylgeranylation of RhoA regulates insulin signalling pathway via TAZ/IRS1 axis

**Figure 5:**
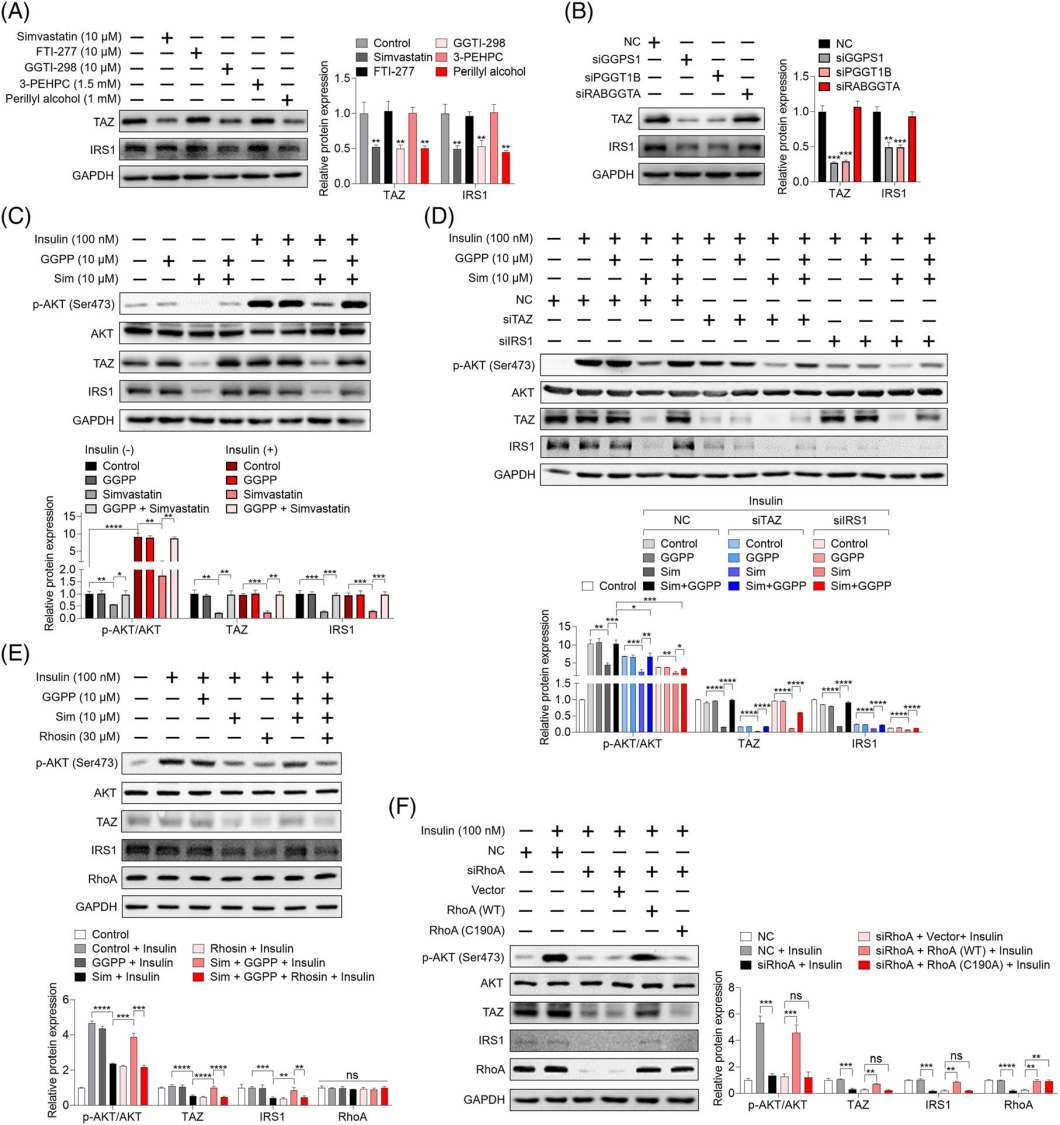
Geranylgeranyl pyrophosphate (GGPP) reverses simvastatin‐caused inhibition of insulin signalling via recovering RhoA geranylgeranylation‐mediated TAZ/IRS1 axis. *(A)* C2C12 myotubes were treated with 10 μM simvastatin, 10 μM FTI‐277, 10 μM GGTI‐298, 1.5 mM 3‐PEHPC, and 1 mM perillyl alcohol for 24 h, protein samples were harvested, and the expression of indicated proteins was analysed by western blot, with GAPDH as the loading control (*n* = 3). *(B)* C2C12 myotubes were transfected with siRNAs specifically targeting GGPS1, PGGT1B, and RABGGTA, respectively, for 48 h. Protein samples were harvested, and the expression of indicated proteins was analysed by western blot, with GAPDH as the loading control (*n* = 3). *(C)* C2C12 myotubes were treated with 10 μM GGPP, 10 μM simvastatin, or 10 μM GGPP combined with 10 μM simvastatin for 24 h. Then cells were treated or not treated with 100 nM insulin for 30 min. Protein samples were harvested, and the expression of indicated proteins was analysed by western blot, with GAPDH as the loading control (*n* = 3). *(D)* C2C12 myotubes were previously transfected with NC or siRNA specifically targeting TAZ and IRS1, respectively, for 48 h. Then cells were treated with 10 μM GGPP, 10 μM simvastatin, or 10 μM GGPP combined with 10 μM simvastatin for another 24 h. Before the end of the experiment, cells were incubated with 100 nM insulin for 30 min. Then protein samples were harvested, and the expression of indicated proteins was analysed by western blot, with GAPDH as the loading control (*n* = 3). *(E)* C2C12 myotubes were treated with 10 μM GGPP, 10 μM simvastatin and 30 μM Rhosin as indicated for 24 h. Then cells were incubated with 100 nM insulin for 30 min, and the expression of indicated proteins was analysed by western blot, with GAPDH as the loading control (*n* = 3). *(F)* C2C12 myotubes were previously transfected with siRNA targeting RhoA for 24 h, and then cells were transfected with vector, RhoA (WT), or RhoA (C190A) plasmids for 48 h. Before the end of the experiment, cells were incubated with 100 nM insulin for 30 min, then total protein samples were harvested, and the expression of indicated proteins was analysed by western blot, with GAPDH as the loading control (*n* = 3). Data represented the mean ± SEM. Statistical analysis was performed with one‐way ANOVA. *P < 0.05; **P < 0.01; ***P < 0.001; ****P < 0.0001.

Next, we set to dissect the mechanism underlying GGPP‐regulated insulin signalling pathway. Based on reported role of TAZ/IRS1 axis in statin‐induced insulin resistance in skeletal muscle,^4^ we hypothesized that TAZ/IRS1 axis might be involved in the beneficial effect of GGPP on insulin signalling. As shown in *Figures* 5A and S9A, in both C2C12 myotubes and primary mouse myotubes, GGTI‐298 and perillyl alcohol mimicked the inhibitory effect of simvastatin on TAZ and IRS1 expression, while FTI‐277 and 3‐PEHPC had no effect. Consistently, knockdown of GGPS1 and PGGT1B inhibited TAZ and IRS1 expression, while RABGGTA knockdown failed (*Figures* 5B and S9B). GGPP reversed simvastatin‐caused inhibition of AKT phosphorylation at Ser473 site, as well as TAZ and IRS1 expression (*Figures* 5C and S9C). In addition, the protective effect of GGPP on insulin signalling and TAZ/IRS1 axis was also verified *in vivo* (*Figure* S10A and S10B). To further study whether GGPP prevented simvastatin‐caused inhibition of AKT phosphorylation at Ser473 site through TAZ/IRS1 axis, knockdown of TAZ or IRS1 using specific siRNAs was performed. siTAZ#3 and siIRS1#3 were used because of their best knockdown efficiency among three designed siRNAs (*Figure* S11A and S11B). As shown in *Figures* 5D and S9C, TAZ and IRS1 knockdown both attenuated the protective effect of GGPP on insulin signalling indicated by decreased phosphorylation of AKT Ser473 site in C2C12 myotubes and primary mouse myotubes. The aforementioned results suggest that GGPP prevents simvastatin‐caused inhibition of insulin signalling through maintaining TAZ/IRS1 axis. Furthermore, it is well documented that TAZ is regulated by RhoA.^17^ Thus, we hypothesized that impaired RhoA geranylgeranylation disrupted insulin signalling via inhibiting TAZ/IRS1 axis. Firstly, we checked the effect of simvastatin and GGTase I inhibition on RhoA geranylgeranylation; it was showed that simvastatin and GGTase I inhibition by GGTI‐298 and PGGT1B knockdown inhibited geranylgeranylation of RhoA in C2C12 myotubes (*Figure* S12A and S12B). Moreover, plasma membrane attachment of RhoA was concomitantly disrupted by simvastatin and GGTase I inhibition in C2C12 myotubes (*Figure* S13A and S13B). GGPP was able to prevent simvastatin‐caused RhoA geranylgeranylation deficiency in C2C12 myotubes (*Figure* S12C), and GGOH was effectively to prevent simvastatin‐caused RhoA geranylgeranylation deficiency in gastrocnemius muscle (*Figure* S12D). These results suggest that geranylgeranylation of RhoA is impaired by simvastatin *in vitro* and *in vivo*. Next, to verify the hypothesis that impaired RhoA geranylgeranylation disrupted insulin signalling via inhibiting TAZ/IRS1 axis, Rhosin, a specific inhibitor of RhoA, was used. As shown in *Figure* 5E, Rhosin inhibited TAZ/IRS1 axis and insulin signalling and attenuated the protective effect of GGPP on simvastatin‐induced inhibition of TAZ/IRS1 axis and insulin signalling in C2C12 myotubes. Besides, RhoA knockdown was performed using specific siRNA targeting RhoA, and siRNA#2 was used based on its best knockdown efficiency among three designed siRNAs (*Figure* S11C). RhoA knockdown inhibited TAZ/IRS1 axis and insulin signalling and also attenuated the protective effect of GGPP on simvastatin‐induced inhibition of TAZ/IRS1 axis and insulin signalling in C2C12 myotubes (*Figure* S14). To study the role of RhoA geranylgeranylation in RhoA‐regulated TAZ/IRS1 axis and insulin signalling in skeletal muscle, site mutation of cysteine to alanine in the CAAX motif located at the carboxyl terminal of RhoA was performed, namely, RhoA (C190A).^33^ The availability of the mutant construct was confirmed by geranylgeranylation deficiency of RhoA (*Figure* S12F). In RhoA knockdown C2C12 myotubes, expression of wild‐type RhoA was sufficient to recover TAZ/IRS1 axis and insulin signalling, but expression of RhoA (C190A) failed (*Figure* 5F). This finding was also confirmed in primary mouse myotubes (*Figure* S9D). Meanwhile, our results also showed that wild‐type RhoA was able to attach to the plasma membrane, but RhoA (C190A) could not (*Figure* S13C). The result suggests that RhoA geranylgeranylation‐mediated attachment to the plasma membrane is necessary for TAZ/IRS1 axis and insulin signalling. Collectively, these data suggest that simvastatin suppresses insulin signalling via disrupting RhoA geranylgeranylation‐mediated TAZ/IRS1 axis.

### Geranylgeranyl pyrophosphate regulates GLUT4 translocation and concomitant glucose uptake via geranylgeranyl transferase II‐mediated geranylgeranylation of RAB8A

**Figure 6:**
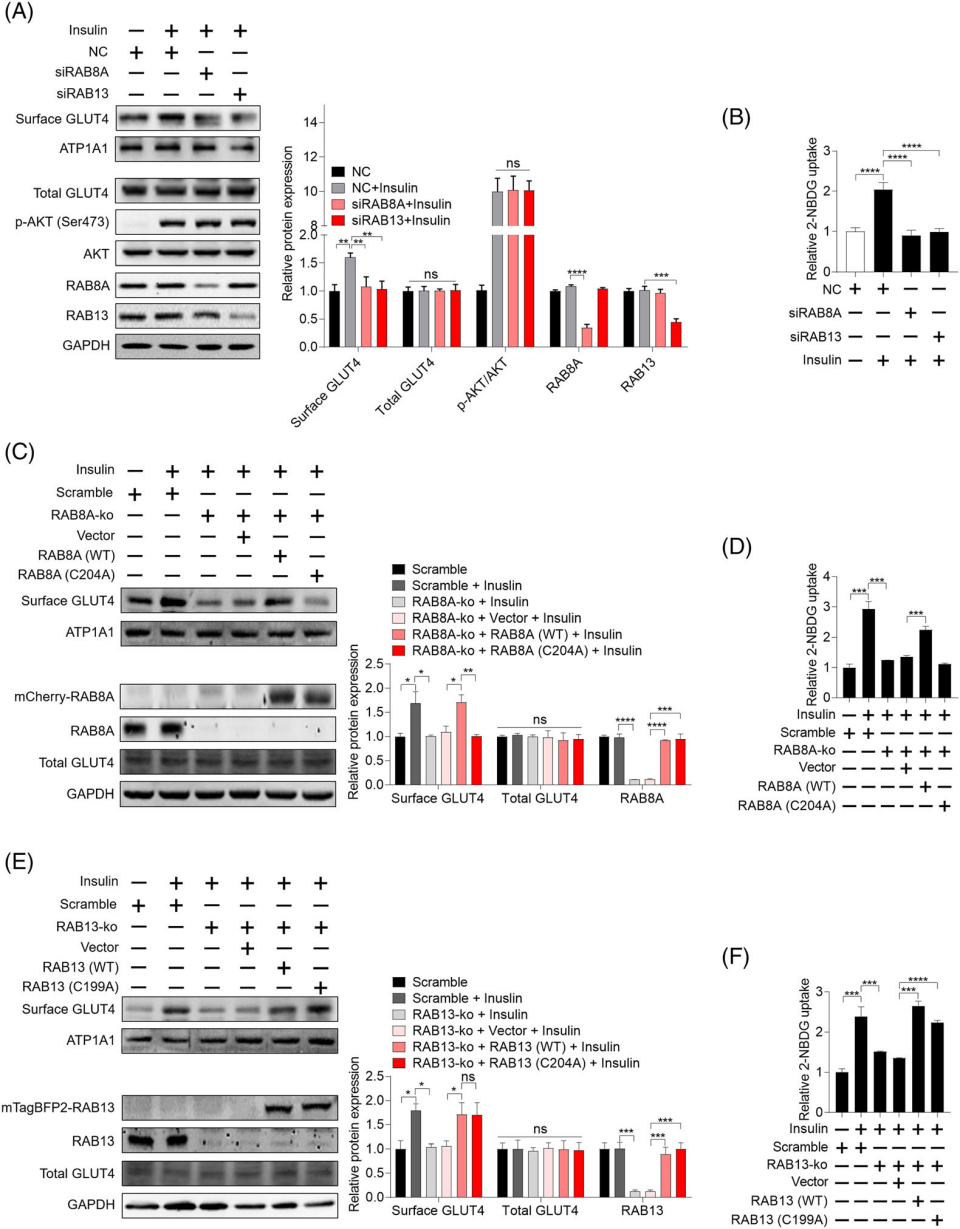
Geranylgeranylation of RAB8A is critical for insulin‐stimulated GLUT4 translocation and concomitant glucose uptake in skeletal muscle cells. *(A)* C2C12 myotubes were transfected with siRNAs specifically targeting RAB8A and RAB13, respectively, for 48 h. Then cells were incubated with 100 nM insulin for 30 min. Total protein samples and plasma membrane fraction samples were harvested, and the expression of indicated proteins was analysed by western blot, with GAPDH as the loading control (*n* = 3). *(B)* C2C12 myotubes were transfected with siRNAs specifically targeting RAB8A and RAB13, respectively, for 48 h. Then cells were exposed to 2‐NBDG containing 100 nM insulin for 30 min, and 2‐NBDG uptake was measured by fluorescence detection (*n* = 3). *(C)* RAB8A‐knockout (RAB8A‐ko) C2C12 myotubes were transfected with vector, RAB8A (WT), and RAB8A (C204A) plasmids for 48 h. Then cells were incubated with 100 nM insulin for 30 min. Total protein samples and plasma membrane fraction samples were harvested, and the expression of indicated proteins was analysed by western blot, with GAPDH as the loading control (*n* = 3). *(D)* RAB8A‐ko C2C12 myotubes were transfected with vector, RAB8A (WT), and RAB8A (C204A) plasmids for 48 h. Then cells were exposed to 2‐NBDG containing 100 nM insulin for 30 min, and 2‐NBDG uptake was measured by fluorescence detection (*n* = 3). *(E)* RAB13‐ko C2C12 myotubes were transfected with vector, RAB13 (WT), and RAB13 (C199A) plasmids for 48 h. Then cells were incubated with 100 nM insulin for 30 min. Total protein samples and plasma membrane fraction samples were harvested, and the expression of indicated proteins was analysed by western blot, with GAPDH as the loading control (*n* = 3). *(F)* RAB13‐ko C2C12 myotubes were transfected with vector, RAB13 (WT), and RAB13 (C199A) plasmids for 48 h. Then cells were exposed to 2‐NBDG containing 100 nM insulin for 30 min, and 2‐NBDG uptake was measured by fluorescence detection (*n* = 3). Data represented the mean ± SEM. Statistical analysis was performed with one‐way ANOVA. *P < 0.05; **P < 0.01; ***P < 0.001; ****P < 0.0001; ns denotes no significance.

Geranylgeranyl transferase II specifically catalyses the geranylgeranylation modification of RAB proteins.^34^ Given the well‐documented role of RAB8A and RAB13 in the insulin‐stimulated mobilization of GSVs,^35^, ^36^ we hypothesized that geranylgeranylation deficiency of RAB8A and RAB13 might impede insulin‐stimulated mobilization of GSVs in skeletal muscle cells. We observed that simvastatin and GGTase II inhibition inhibited geranylgeranylation of RAB8A and RAB13 (*Figure* S12A, S12B, and S12E). In addition, GGPP was able to prevent simvastatin‐caused geranylgeranylation deficiency of RAB8A and RAB13 in C2C12 myotubes and GGOH prevented simvastatin‐triggered geranylgeranylation deficiency of RAB8A and RAB13 in gastrocnemius muscle (*Figure* S12C and S12D). Furthermore, knockdown of RAB8A and RAB13 was performed using specific siRNAs; siRAB8A#2 and siRAB13#2 were picked on account of their highest knockdown efficiency among three designed siRNAs (*Figure* S15A and S15B). As shown in *Figures* 6A, 6B, S16A, and S16B, knockdown of RAB8A or RAB13 both suppressed insulin‐stimulated GLUT4 translocation and concomitant glucose uptake, without disturbing insulin signalling and total GLUT4 expression in C2C12 myotubes and primary mouse myotubes. To further confirm these findings, RAB8A‐knockout (RAB8A‐ko) C2C12 myoblasts and RAB13‐knockout (RAB13‐ko) myoblasts were established using lentivirus‐mediated CRISPR‐Cas 9 system (*Figure* S15C and S15D). RAB8A knockdown or RAB13 knockdown‐elicited suppressed insulin‐stimulated GLUT4 translocation and concomitant glucose uptake was completely repeated by RAB8A knockout or RAB13 knockout in C2C12 myotubes (*Figure* S17A and S17B). To test whether geranylgeranylation deficiency of RAB8A and RAB13 lead to disrupted GLUT4 translocation and glucose uptake, mouse RAB8A and RAB13 with mutation of Cys site in the carboxyl terminal were constructed, namely, RAB8A (C204A) and RAB13 (C199A).^34^ The availability of these two mutant constructs was confirmed by geranylgeranylation deficiency (*Figure* S12G and S12H). In RAB8A‐ko C2C12 myotubes, expression of wild‐type RAB8A, namely, RAB8A (WT), was sufficient to restore insulin‐stimulated GLUT4 translocation and concomitant glucose uptake, but RAB8A (C204A) failed to do so (*Figure* 6C and 6D). In RAB13‐ko C2C12 myotubes, both wild‐type RAB8A, namely, RAB13 (WT), and RAB13 (C199A) were sufficient to restore insulin‐stimulated GLUT4 translocation and concomitant glucose uptake (*Figure* 6E and 6F). Similarly, in primary mouse myotubes with RAB8A knockdown, expression of RAB8A (WT), but not RAB8A (C204A), was able to restore insulin‐stimulated GLUT4 translocation and concomitant glucose uptake (*Figure* S16C and S16D). In primary mouse myotubes with RAB13 knockdown, both RAB13 (WT) and RAB13 (C199A) were able to restore insulin‐stimulated GLUT4 translocation and concomitant glucose uptake (*Figure* S16E and S16F). Taken together, the aforementioned data suggest that RAB8A geranylgeranylation is necessary for insulin‐stimulated GLUT4 translocation in skeletal muscle cells.

## Discussion

**Figure 7:**
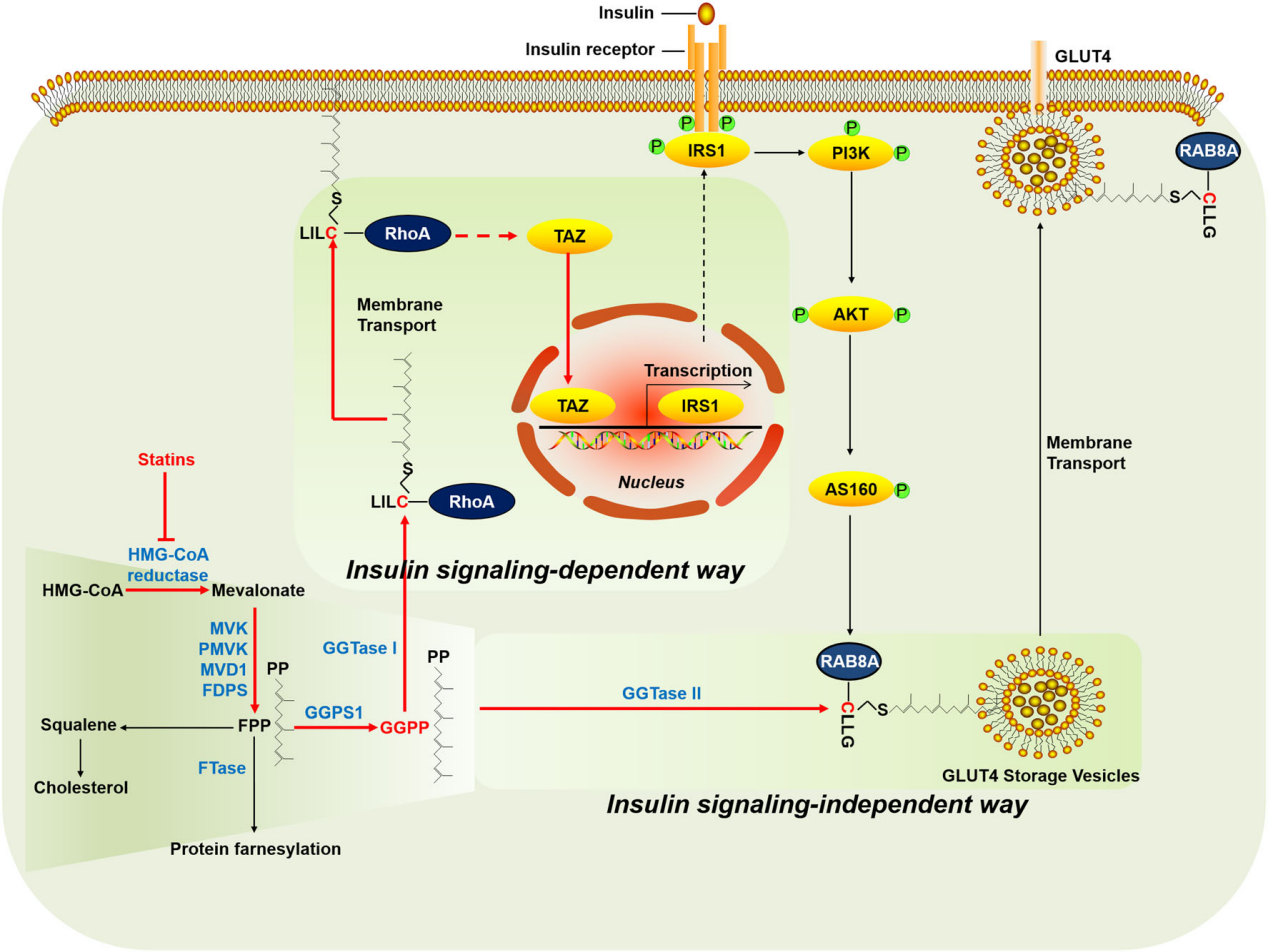
Schematic diagram of proposed statin‐targeted mevalonate pathway regulating insulin‐stimulated glucose uptake.

The present study has demonstrated that statins induce insulin resistance in skeletal muscle by inhibiting GGPP production. GGTase I modulates insulin sensitivity in skeletal muscle in a GGPP/RhoA/TAZ/IRS1 pathway‐mediated insulin signalling‐dependent manner, while GGTase II is directly involved in the plasma membrane localization of GLUT4 through geranylgeranylation of RAB8A, regulating insulin sensitivity in skeletal muscle in an insulin signalling‐independent way (*Figure* 7).

In order to verify the effect of statins on new‐onset diabetes in mice, simvastatin and lovastatin with the strongest lipophicity were selected. Because statins are used in patients with abnormal lipid metabolism, we investigated the effect of statins in normal condition and abnormal lipid metabolism condition on new‐onset diabetes in mice. High‐dose simvastatin (80 mg/kg/day) fed for 8 weeks directly increased fasting glucose in C57BL/6J mice and significantly impaired glucose tolerance and insulin tolerance as an indicator of insulin sensitivity. In obese mice induced by a high‐fat diet, high‐dose lovastatin (125 mg/kg/day) fed for 8 weeks also directly increased fasting glucose in obese mice, as well as impaired glucose and insulin tolerance. These results strongly support the conclusion that statins cause new‐onset diabetes. Insulin resistance is an earlier stage in the development of type 2 diabetes, and skeletal muscle, as the largest glucose consumption and insulin‐sensitive organ in the human body, is responsible for the disposal of ~2/3 of postprandial blood glucose.^10^, ^37^ Therefore, it is of great significance to study the molecular mechanism of insulin resistance in skeletal muscle induced by statins. Although Hwang *et al*. have reported that the TAZ/IRS1 axis mediates statin‐induced skeletal muscle insulin resistance,^4^ whether and how GGPP is involved in statin‐induced skeletal muscle insulin resistance remains unclear. In addition, the Hwang *et al*. model of statin‐induced insulin resistance in mice was also adopted in this study; namely, normal C57BL/6J male mice were fed with simvastatin (40 mg/kg/day) for 3 weeks.

In this study, chemical inhibitors were used to investigate the effects of FTase, GGTase I, and GGTase II inhibition on insulin‐stimulated glucose uptake. This experiment ruled out a role for FPP in statin‐induced skeletal muscle insulin resistance.

Then GGTase I and GGTase II were inhibited by siRNA‐mediated gene interference, with GGPS1 knockdown as the positive control to strengthen the hypothesis that GGPP depletion was the inducement of insulin resistance in skeletal muscle caused by statins. By replenishing GGPP, the decrease of insulin‐induced glucose uptake in skeletal muscle cells induced by simvastatin and GGPS1 knockdown was attenuated, thus demonstrating that GGPP depletion was the cause of statin‐induced insulin resistance in skeletal muscle. Obviously, this conclusion is contrary to the Sun *et al*. report that cholesterol depletion disrupts glucose uptake in skeletal muscle cells.^38^ We therefore investigated whether cholesterol could reverse simvastatin‐induced inhibition of insulin‐stimulated glucose uptake in skeletal muscle cells. The results showed that cholesterol could not reverse the inhibition of insulin‐stimulated glucose uptake in skeletal muscle cells, which may be caused by different experimental conditions.

The regulatory effect of GGPP on insulin sensitivity in skeletal muscle cells was directly demonstrated by using chemical inhibitors and siRNA‐mediated gene interference to inhibit the GGPP transferases responsible for the covalent binding of GGPP to target proteins. Our data shown that GGTase I inhibition impaired geranylgeranylation of RhoA and down‐regulated the expression of TAZ and IRS1, thus inhibiting insulin signalling. To our surprise, GGTase II inhibition did not affect insulin signalling and the expression of TAZ and IRS1, demonstrating that GGTase II inhibition suppressed insulin‐stimulated GLUT4 membrane transport and concomitant glucose uptake in skeletal muscle cells in an insulin signalling‐independent manner. This is a novel finding; thus, AAV9‐mediated *in vivo* knockdown of skeletal muscle RABGGTA, the specific subunit of GGTase II, was performed to verify this finding *in vivo*. Consistently, RABGGTA knockdown‐mediated GGTase II inhibition in skeletal muscle caused insulin resistance without disturbing insulin signalling *in vivo*. GGTase II is specifically responsible for geranylgeranylation of the RAB protein family. Based on present studies, RAB8A and RAB13 were well‐established RAB proteins involved in intracellular transport of GSVs in skeletal muscle^39^; thus, RAB8A and RAB13 were selected as the object of investigation. The function of geranylgeranylation of RAB8A and RAB13 was investigated by site mutation of the geranylgeranylation modification site, the cysteine site at the carboxyl terminal of RAB8A and RAB13. The data showed that geranylgeranylation deficiency of RAB8A reappeared RAB8A knockdown‐induced inhibition of insulin‐stimulated GLUT4 translocation and concomitant glucose uptake. Unexpectedly, geranylgeranylation deficiency of RAB13 did not show any influence on insulin‐stimulated GLUT4 translocation and concomitant glucose uptake. This was consistent with the Ioannou *et al*. report that RAB13 traffics on vesicles independent of prenylation.^40^ In addition, RAB13 has more colocalization with GLUT4 at the perinuclear area, whereas RAB8A has colocalization with GLUT4 at both the perinuclear compartments and the periphery of the cells.^41^ Thus, RAB8A is more likely to play as a ‘transport machine’ for the transport of GSVs from the perinuclear area to the plasma membrane.

There were also some limitations in our study. RAB8A and RAB13 were chosen as the candidate just based on present research status. However, the involvement of other members of the RAB protein family cannot be ruled out. In addition, insertion of the carboxyl‐terminal geranylgeranyl group into the membrane was not visualized in our study. Thus, whether RAB8A regulates the mobilization of GSVs via insertion of the carboxyl‐terminal geranylgeranyl group into the membrane of GSVs is still needed to be elucidated in our future studies. In conclusion, our results uncovered that statins inhibit GGPP production and induce insulin resistance in skeletal muscle cells through RhoA geranylgeranylation‐mediated insulin signalling‐dependent way and RAB8A geranylgeranylation‐mediated insulin signalling‐independent way. Supplementation of GGOH effectively protected against statin‐caused inhibition of glucose disposal in skeletal muscle and whole‐body insulin resistance.

## Funding

This work was supported by the National Key R&D Program of China (2019YFC1711000).

## Conflict of interest

Lai Wang, Zuguo Zheng, Lijun Zhu, Lingchang Meng, Hanling Liu, Keke Wang, Jun Chen, Ping Li, and Hua Yang declare that they have no conflict of interest.

## Supporting information




**Figure S1** Evaluation of the diabetogenic effect of statins *in vivo* and *in vitro*. Statins induced insulin resistance *in vivo* and *in vitro*. Male C57BL/6J mice (20 ± 2 g) were randomly grouped (*n* = 10 for control group and *n* = 9 for simvastatin group). Mice in simvastatin group were intragastrically administrated with simvastatin (80 mg/kg/day) for 8 weeks and mice in control group were given 0.5% CMC‐Na solution. At the end of the experiment, mice were subjected with experiments as follows. (A) Fasted blood glucose level. (B) GTT and GTT AUC. (C) ITT and ITT AUC. High‐fat diet‐induced obese male C57BL/6J mice were were randomly grouped (*n* = 5). Mice in lovastatin group were intragastrically administrated with lovastatin (125 mg/kg/day) for 8 weeks and mice in control group were given 0.5% CMC‐Na solution. At the end of the experiment, mice were subjected with experiments as follows. (D) Fasting blood glucose level. (E) GTT and GTT AUC. (F) ITT and ITT AUC. (G) C2C12 myotubes and primary mouse myotubes were treated with 10 μM various statins for 24 h, then cells were exposed to 2‐NBDG containing 100 nM insulin for 30 min and 2‐NBDG uptake was measured by fluorescence detection (*n* = 5). (H) C2C12 myotubes were pretreated with simvastatin (2 μM, 10 μM, 50 μM) for 24 h, then cells were exposed to 2‐NBDG containing 100 nM insulin for 30 min and 2‐NBDG uptake was measured by fluorescence detection (*n* = 5). Data represented the mean ± SEM. Statistical analysis was done with one‐way ANOVA. *P < 0.05; **P < 0.01; ***P < 0.001; ****P < 0.0001.

**Figure S2** Knockdown of GGPS1, PGGT1B and RABGGTA suppressed insulin‐stimulated glucose uptake in C2C12 myotubes. C2C12 myoblasts were transfected with siRNAs targeting GGPS1 (A), PGGT1B (B) and RABGGTA (C) using Lipofectamine 3000 for 48 h. Protein samples were harvested and the knockdown efficiency was checked by western blot, with GAPDH as the loading control (*n* = 3). (D) C2C12 myotubes were previously transfected with siRNAs targeting GGPS1, PGGT1B and RABGGTA respectively for 48 h, then cells were exposed to 2‐NBDG containing 100 nM insulin for 30 min and 2‐NBDG uptake was measured by fluorescence detection (*n* = 3). (E) C2C12 myotubes were previously transfected with siRNA targeting GGPS1 for 24 h, then cells were treated or not treated with 10 μM GGPP for another 24 h. Cells were exposed to 2‐NBDG containing 100 nM insulin for 30 min and 2‐NBDG uptake was measured by fluorescence detection (*n* = 3). Data represented the mean ± SEM. Statistical analysis was done with one‐way ANOVA. **P < 0.01; ****P < 0.0001.

**Figure S3** Lipophilic statins suppress insulin sensitivity *via* inhibiting GGPP production, not cholesterol. (A) C2C12 myotubes were pretreated with 10 μM GGPP, 10 μM GGTI‐298 and 10 μM GGPP combined with 10 μM GGTI‐298 for 24 h, then cells were exposed to 2‐NBDG containing 100 nM insulin for 30 min and 2‐NBDG uptake was measured by fluorescence detection (*n* = 5). (B) C2C12 myotubes were pretreated with 10 μM GGPP, 1.5 mM 3‐PEHPC and 10 μM GGPP combined with 1.5 mM 3‐PEHPC for 24 h, then cells were exposed to 2‐NBDG containing 100 nM insulin for 30 min and 2‐NBDG uptake was measured by fluorescence detection (*n* = 5). (C) C2C12 myotubes were pretreated with 10 μM GGPP, 1 mM perillyl alcohol and 10 μM GGPP combined with 1 mM perillyl alcohol for 24 h, then cells were exposed to 2‐NBDG containing 100 nM insulin for 30 min and 2‐NBDG uptake was measured by fluorescence detection (*n* = 5). (D) C2C12 myotubes were previously transfected with siRNA targeting PGGT1B for 24 h, then cells were treated or not treated with 10 μM GGPP for another 24 h. cells were exposed to 2‐NBDG containing 100 nM insulin for 30 min and 2‐NBDG uptake was measured by fluorescence detection (*n* = 3). (E) C2C12 myotubes were previously transfected with siRNA targeting RABGGTA for 24 h, then cells were treated or not treated with 10 μM GGPP for another 24 h. cells were exposed to 2‐NBDG containing 100 nM insulin for 30 min and 2‐NBDG uptake was measured by fluorescence detection (*n* = 3). (F) C2C12 myotubes were pretreated with 10 μM MβCD‐cholesterol, 10 μM simvastatin and 10 μM MβCD‐cholesterol combined with 10 μM simvastatin for 24 h, then cells were exposed to 2‐NBDG containing 100 nM insulin for 30 min and 2‐NBDG uptake was measured by fluorescence detection (*n* = 5). Data represented the mean ± SEM. Statistical analysis was done with one‐way ANOVA. *P < 0.05; **P < 0.01; ***P < 0.001; ****P < 0.0001.

**Figure S4** Enhanced insulin sensitivity by GGOH treatment could not be attributed to differences in insulin secretion, muscle mass and muscle fibre type composition. Male C57BL/6J mice (20 ± 2 g) were randomly grouped (*n* = 6). After administration of GGOH (25 mg/kg/day), simvastatin (40 mg/kg/day), and GGOH combined with simvastatin for 3 weeks, mice were subjected with experiments below. (A) Body weight. (B) Serum insulin levels was measured using ELISA kit. (C) HE staining of the cross section of gastrocnemius. The representative pictures were shown. (D) Wet weight of gastrocnemius, tibialis anterior, soleus and extensor digitus longus. (E) The expression of Myh7, Myh2, Myh4 and Myh1 in gastrocnemius, tibialis anterior, soleus and extensor digitus longus was analyzed by RT‐qPCR. Data represented the mean ± SEM. Statistical analysis was done with one‐way ANOVA. ns meant no significance.

**Figure S5** (A) C2C12 myoblasts expressing eGFP‐GLUT4 was pretreated with 10 μM simvastatin, 10 μM FTI‐277, 10 μM GGTI‐298, 1.5 mM 3‐PEHPC and 1 mM perillyl alcohol for 24 h. Then cells were incubated with 100 nM insulin for 30 min, localization of GLUT4 was captured by fluorescence confocal microscope (*n* = 3). (B) C2C12 myoblasts expressing eGFP‐GLUT4 were previously transfected with siRNAs targeting GGPS1, PGGT1B and RABGGTA respectively for 48 h, then cells were treated with 100 nM insulin for 30 min, localization of GLUT4 was captured by fluorescence confocal microscope (*n* = 3). (C) Cells were incubated with 100 nM insulin for 30 min and localization of GLUT4 was captured by fluorescence confocal microscope (*n* = 3). (D) Cells were incubated with 100 nM insulin for 30 min and localization of GLUT4 was captured by fluorescence confocal microscope (n = 3). The fluorescence intensity was analyzed using ImageJ.

**Figure S6** Insulin signaling is not necessary for simvastatin‐caused inhibition of insulin stimulated‐glucose uptake in skeletal muscle cells. (A) Primary mouse myotubes were pretreated with 10 μM simvastatin, 10 μM FTI‐277, 10 μM GGTI‐298, 1.5 mM 3‐PEHPC and 1 mM perillyl alcohol for 24 h. Then cells were incubated with or without 100 nM insulin for 30 min. Total protein was harvested and the expression of indicated proteins was analyzed by western blot, with GAPDH as the loading control (*n* = 3). (B) Primary mouse myotubes were previously transfected with siRNAs targeting GGPS1, PGGT1B and RABGGTA respectively for 48 h. Then cells were incubated with or without 100 nM insulin for 30 min. Total protein was harvested and the expression of indicated proteins was analyzed by western blot, with GAPDH as the loading control (*n* = 3). Data represented the mean ± SEM. Statistical analysis was done with one‐way ANOVA. **P < 0.01; ***P < 0.001; ****P < 0.0001; ns meant no significance.

**Figure S7** Myr‐AKT reversed the inhibitory effect of simvastatin on insulin signaling. (A) C2C12 myotubes were transfected with 1 μg Myr‐AKT plasmid using Lipofectamine 3000 for 48 h. Protein samples were harvested and the expression of indicated proteins was checked by western blot, with GAPDH as the loading control (*n* = 3). (B) C2C12 myotubes were previously transfected with or without 1 μg Myr‐AKT plasmid using Lipofectamine 3000 for 48 h, then the cells were treated with or without 10 μM simvastatin for another 24 h. Before the end of the experiment, cells were incubated with or without 100 nM insulin for 30 min, then protein samples were harvested and the expression of indicated proteins was checked by western blot, with GAPDH as the loading control (*n* = 3). Data represented the mean ± SEM. Statistical analysis was done with one‐way ANOVA. *P < 0.05; **P < 0.01; ns meant no significance.

**Figure S8** RABGGTA knockdown‐induced insulin resistance could not be attributed to differences in insulin secretion, muscle mass and muscle fibre type composition. Mice were subjected a week of adjustable feeding, then were divided into two groups including shControl group and shRABGGTA group (*n* = 6). Posterior limbs of mice in shControl group and shRABGGTA group were infected with control AAV9 and shRABGGTA AAV9 respectively through in situ injection. 4 weeks after the infection, mice were sacrificed and subjected to a series of analyzes as indicated below. (A) *Invivo* imaging of infected posterior limbs. (B) Body weight. (C) Serum insulin levels. (D) HE staining of the cross section of gastrocnemius. The representative pictures were shown. (E) The wet weights of the gastrocnemius, soleus, tibialis anterior and extensor digitorum longus muscles. (F) Transcript levels of Myh7, Myh2, Myh4 and Myh1 in gastrocnemius, tibialis anterior, soleus and extensor digitorum longus muscles were analyzed by RT‐qPCR to determine the composition of muscle fibre types. Data represented the mean ± SEM. Statistical analysis was done with one‐way ANOVA. ns meant no significance.

**Figure S9** Simvastatin modulated insulin signaling *via* RhoA geranylgeranylation‐mediated TAZ/IRS1 axis in primary mouse myotubes. (A) Primary mouse myotubes were treated with 10 μM simvastatin, 10 μM FTI‐277, 10 μM GGTI‐298, 1.5 mM 3‐PEHPC and 1 mM perillyl alcohol for 24 h, protein samples were harvested and the expression of indicated proteins was analyzed by western blot, with GAPDH as the loading control (*n* = 3). (B) Primary mouse myotubes were transfected with siRNAs specifically targeting GGPS1, PGGT1B and RABGGTA respectively for 48 h. Protein samples were harvested and the expression of indicated proteins was analyzed by western blot, with GAPDH as the loading control (n = 3). (C) Primary mouse myotubes was previously transfected with NC or siRNA specifically targeting TAZ and IRS1 respectively for 48 h. Then cells were treated with 10 μM GGPP, 10 μM simvastatin or 10 μM GGPP combined with 10 μM simvastatin for another 24 h. Before the end of the experiment, cells were incubated with 100 nM insulin for 30 min. Then protein samples were harvested and the expression of indicated proteins were analyzed by western blot, with GAPDH as the loading control (*n* = 3). (D) Primary mouse myotubes were previously transfected with siRNA targeting RhoA for 24 h, then cells were transfected with vector, RhoA (WT) or RhoA (C190A) plasmids for 48 h. Before the end of the experiment, cells were incubated with 100 nM insulin for 30 min, then total protein samples were harvested and the expression of indicated proteins was analyzed by western blot, with GAPDH as the loading control (*n* = 3). Data represented the mean ± SEM. Statistical analysis was done with one‐way ANOVA. *P < 0.05; **P < 0.01; ***P < 0.001; ****P < 0.0001.

**Figure S10** GGOH impeded the inhibition of simvastatin on insulin signaling and TAZ/IRS1 axis in skeletal muscle. *(A, B)* Male C57BL/6J mice (20 ± 2 g) were randomly grouped (*n* = 6). After administration of GGOH (25 mg/kg/day), simvastatin (40 mg/kg/day), and GGOH combined with simvastatin for 3 weeks, mice were sacrificed and the expression of indicated proteins in gastrocnemius *(A)* and tibialis anterior *(B)* was analyzed by western blot, with GAPDH as the loading control (*n* = 3). Data represented the mean ± SEM. Statistical analysis was done with one‐way ANOVA. *P < 0.05; **P < 0.01; ***P < 0.001.

**Figure S11** Screening effective siRNA sequences for the knockdown of TAZ, IRS1 and RhoA. C2C12 myoblasts were transfected with siRNAs targeting TAZ (A), IRS1 (B) and RhoA (C) using Lipofectamine 3000 for 48 h. Protein samples were harvested and the knockdown efficiency was checked by western blot, with GAPDH as the loading control (*n* = 3). Data represented the mean ± SEM. Statistical analysis was done with one‐way ANOVA. *P < 0.05; **P < 0.01; ***P < 0.001.

**Figure S12** Geranylgeranylation of RhoA, RAB8A and RAB13 was analyzed in C2C12 myotubes and gastrocnemius muscle. (A‐C, F‐H) C2C12 myotubes were treated with 10 μM simvastatin, 10 μM GGTI‐298, 1.5 mM 3‐PEHPC for 24 h (A), C2C12 myotubes were transfected with siRNAs targeting GGPS1, PGGT1B and RABGGTA using Lipofectamine 3000 for 48 h (B), C2C12 myotubes were pretreated with 10 μM GGPP, 10 μM simvastatin and 10 μM GGPP combined with 10 μM simvastatin for 24 h (C), C2C12 myotubes were previously transfected with siRNA targeting RhoA for 24 h, then cells were transfected with vector, RhoA (WT) or RhoA (C190A) plasmids for 48 h (F), RAB8A‐ko C2C12 myotubes were transfected with vector, RAB8A (WT) and RAB8A (C204A) plasmids for 48 h (G), RAB13‐ko C2C12 myotubes were transfected with vector, RAB13 (WT) and RAB13 (C199A) plasmids for 48 h (H). Unprocessed RhoA, RAB8A, RAB13 and geranylgeranylated RhoA, RAB8A, RAB13 in these samples were separated by the Triton X‐114 partition method and analyzed by western blot (*n* = 3). (D, E) Male C57BL/6J mice (20 ± 2 g) were randomly grouped (*n* = 6). After administration of GGOH (25 mg/kg/day), simvastatin (40 mg/kg/day), and GGOH combined with simvastatin for 3 weeks. 10 mg gastrocnemius muscle tissue was incised from every mouse. Gastrocnemius muscle tissues from one group were mixed (n = 6) (D). Mice were subjected a week of adjustable feeding, then were divided into two groups including shControl group and shRABGGTA group (n = 6). Posterior limbs of mice in shControl group and shRABGGTA group were infected with control AAV9 and shRABGGTA AAV9 respectively through in situ injection for 4 weeks. 20 mg gastrocnemius muscle tissue was incised from mice (*n* = 3). Unprocessed RhoA, RAB8A, RAB13 and geranylgeranylated RhoA, RAB8A, RAB13 in gastrocnemius muscle tissues were separated by the Triton X‐114 partition method and analyzed by western blot.

**Figure S13** Pharmaceutical and genetic inhibition of GGTase I inhibited the attachment of RhoA to plasma membrane. (A) C2C12 myotubes were treated with 10 μM simvastatin, 10 μM GGTI‐298 and 1.5 mM 3‐PEHPC respectively for 24 h. Before the end of the experiment, cells were incubated with 100 nM insulin for 30 min, then cells were harvested and membrane fractions were extracted. RhoA expression in membrane fraction was analyzed by western blot, with GAPDH as the loading control (*n* = 3). (B) C2C12 myotubes were transfected with siRNAs targeting PGGT1B and RABGGTA respectively using Lipofectamine 3000 for 48 h. Before the end of the experiment, cells were incubated with 100 nM insulin for 30 min, then cells were harvested and membrane fractions were extracted. RhoA expression in membrane fraction was analyzed by western blot, with GAPDH as the loading control (*n* = 3). (C) C2C12 myotubes were previously transfected with siRNA targeting RhoA for 24 h, then cells were transfected with vector, RhoA (WT) or RhoA (C190A) plasmids for 48 h. Before the end of the experiment, cells were incubated with 100 nM insulin for 30 min, then plasma membrane protein samples were harvested and the expression of indicated proteins was analyzed by western blot, with GAPDH as the loading control (n = 3). Data represented the mean ± SEM. Statistical analysis was done with one‐way ANOVA. **P < 0.01; ***P < 0.001; ns meant no significance.

**Figure S14** RhoA knockdown attenuated the protective effect of GGPP on simvastatin‐caused inhibition of TAZ/IRS1 axis and insulin signaling. C2C12 myotubes were previously transfected with siRNA targeting RhoA for 48 h, then cells were treated with 10 μM GGPP, 10 μM simvastatin and 10 μM GGPP combined with 10 μM simvastatin respectively for another 24 h. Before the end of the experiment, cells were incubated with 100 nM insulin for 30 min. Protein samples were harvested and the expression of indicated proteins were analyzed by western blot, with GAPDH as the loading control (*n* = 3). Data represented the mean ± SEM. Statistical analysis was done with one‐way ANOVA. **P < 0.01; ***P < 0.001.

**Figure S15** Screening effective siRNA and sgRNA sequences for the knockdown and knockout of RAB8A and RAB13. (A, B) C2C12 myoblasts were transfected with siRNAs targeting RAB8A (A), rab13 (B) using Lipofectamine 3000 for 48 h. Protein samples were harvested and the knockdown efficiency was checked by western blot, with GAPDH as the loading control (*n* = 3). (C, D) C2C12 myoblasts were previously transfected with lentivirus packaged Cas 9 expressing plasmid DNA for 72 h to establish Cas 9‐expressing C2C12 myoblasts. Then Cas 9‐expressing C2C12 myoblasts were transfected with lentivirus packaged RAB8A sgRNAs (C) or RAB13 sgRNAs (D) for 72 h. Protein samples were harvested and the knockout efficiency was analyzed by western blot, with GAPDH as the loading control (n = 3). Data represented the mean ± SEM. Statistical analysis was done with one‐way ANOVA. *P < 0.05; **P < 0.01; ***P < 0.001; ****P < 0.0001.

**Figure S16** Geranylgeranylation of RAB8A was critical for insulin‐stimulated GLUT4 translocation and concomitant glucose uptake in skeletal muscle cells. *(A)* Primary mouse myotubes were transfected with siRNAs specifically targeting RAB8A and RAB13 respectively for 48 h. Then cells were incubated with 100 nM insulin for 30 min. Total protein samples and plasma membrane fraction samples were harvested and the expression of indicated proteins were analyzed by western blot, with GAPDH as the loading control (*n* = 3). *(B)* Primary mouse myotubes were transfected with siRNAs specifically targeting RAB8A and RAB13 respectively for 48 h. Then cells were exposed to 2‐NBDG containing 100 nM insulin for 30 min and 2‐NBDG uptake was measured by fluorescence detection (*n* = 3). *(C)* Primary mouse myotubes were previously transfected with RAB8A siRNA for 24 h for RAB8A knockdown. Then these cells were transfected with vector, RAB8A (WT) and RAB8A (C204A) plasmids for another 48 h. Next, cells were incubated with 100 nM insulin for 30 min. Total protein samples and plasma membrane fraction samples were harvested and the expression of indicated proteins were analyzed by western blot, with GAPDH as the loading control (*n* = 3). *(D)* Primary mouse myotubes were previously transfected with RAB8A siRNA for 24 h for RAB8A knockdown. Then these cells were transfected were transfected with vector, RAB8A (WT) and RAB8A (C204A) plasmids for 48 h. Next, cells were exposed to 2‐NBDG containing 100 nM insulin for 30 min and 2‐NBDG uptake was measured by fluorescence detection (*n* = 3). *(E)* Primary mouse myotubes were previously transfected with RAB13 siRNA for 24 h for RAB8A knockdown. Then these cells were transfected with vector, RAB13 (WT) and RAB13 (C199A) plasmids for another 48 h. Next, cells were incubated with 100 nM insulin for 30 min. Total protein samples and plasma membrane fraction samples were harvested and the expression of indicated proteins were analyzed by western blot, with GAPDH as the loading control (*n* = 3). *(F)* Primary mouse myotubes were previously transfected with RAB8A siRNA for 24 h for RAB13 knockdown. Then these cells were transfected were transfected with vector, RAB13 (WT) and RAB13 (C199A) plasmids for 48 h. Next, cells were exposed to 2‐NBDG containing 100 nM insulin for 30 min and 2‐NBDG uptake was measured by fluorescence detection (n = 3). Data represented the mean ± SEM. Statistical analysis was done with one‐way ANOVA. *P < 0.05; **P < 0.01; ***P < 0.001; ****P < 0.0001; ns meant no significance.

**Figure S17** RAB8A knockout and RAB13 knockout suppressed insulin stimulated GLUT4 translocation and concomitant glucose uptake without disturbing insulin signaling. (A) Scramble, RAB8A‐ko and RAB13‐ko C2C12 myotubes were incubated with 100 nM insulin for 30 min. Total protein samples and membrane fraction samples were harvested and the expression of indicated proteins was analyzed by western blot, with GAPDH as the loading control (*n* = 3). (B) Scramble, RAB8A‐ko and RAB13‐ko C2C12 myotubes were exposed to 2‐NBDG containing 100 nM insulin for 30 min and 2‐NBDG uptake was measured by fluorescence detection (*n* = 5). Data represented the mean ± SEM. Statistical analysis was done with one‐way ANOVA. **P < 0.01; ***P < 0.001; ****P < 0.0001; ns meant no significance.

**Data S1.** Supporting Information
