# Supplementary figures and images for: Geranylgeranyl pyrophosphate depletion by statins compromises skeletal muscle insulin sensitivity
Source: J Cachexia Sarcopenia Muscle. 2022 Aug 12;13(6):2697–711. doi: 10.1002/jcsm.13061 (PMC9745480; doi:10.1002/jcsm.13061)

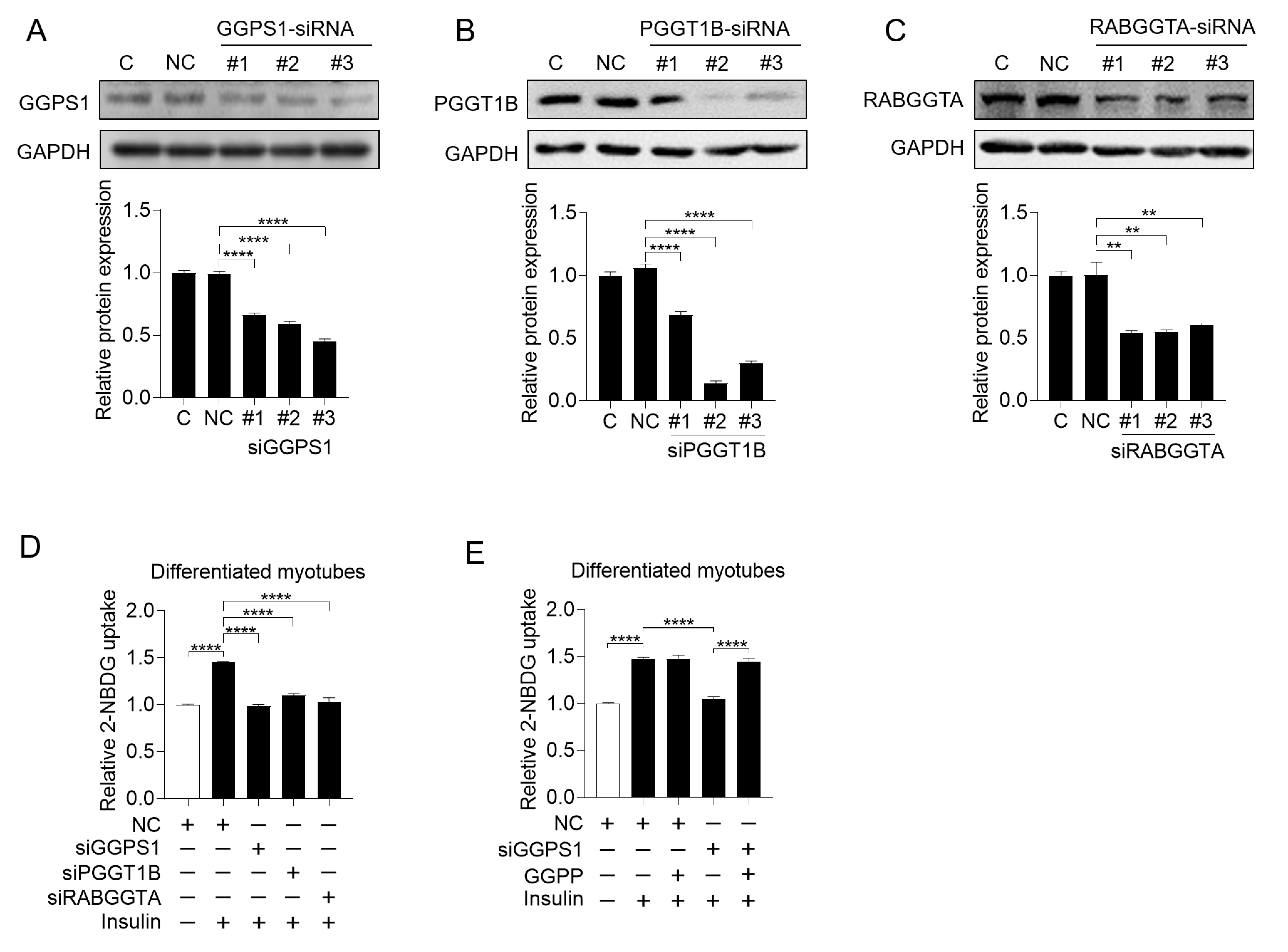

Supplement: Supplementary file 2 — Figure S2 Knockdown of GGPS1, PGGT1B and RABGGTA suppressed insulin‐stimulated glucose uptake in C2C12 myotubes. C2C12 myoblasts were transfected with siRNAs targeting GGPS1 (A), PGGT1B (B) and RABGGTA (C) using Lipofectamine 3000 for 48 h. Protein samples were harvested and the knockdown efficiency was checked by western blot, with GAPDH as the loading control (n = 3). (D) C2C12 myotubes were previously transfected with siRNAs targeting GGPS1, PGGT1B and RABGGTA respectively for 48 h, then cells were exposed to 2‐NBDG containing 100 nM insulin for 30 min and 2‐NBDG uptake was measured by fluorescence detection (n = 3). (E) C2C12 myotubes were previously transfected with siRNA targeting GGPS1 for 24 h, then cells were treated or not treated with 10 μM GGPP for another 24 h. Cells were exposed to 2‐NBDG containing 100 nM insulin for 30 min and 2‐NBDG uptake was measured by fluorescence detection (n = 3). Data represented the mean ± SEM. Statistical analysis was done with one‐way ANOVA. **P < 0.01; ****P < 0.0001. [file JCSM-13-2697-s015.tif]

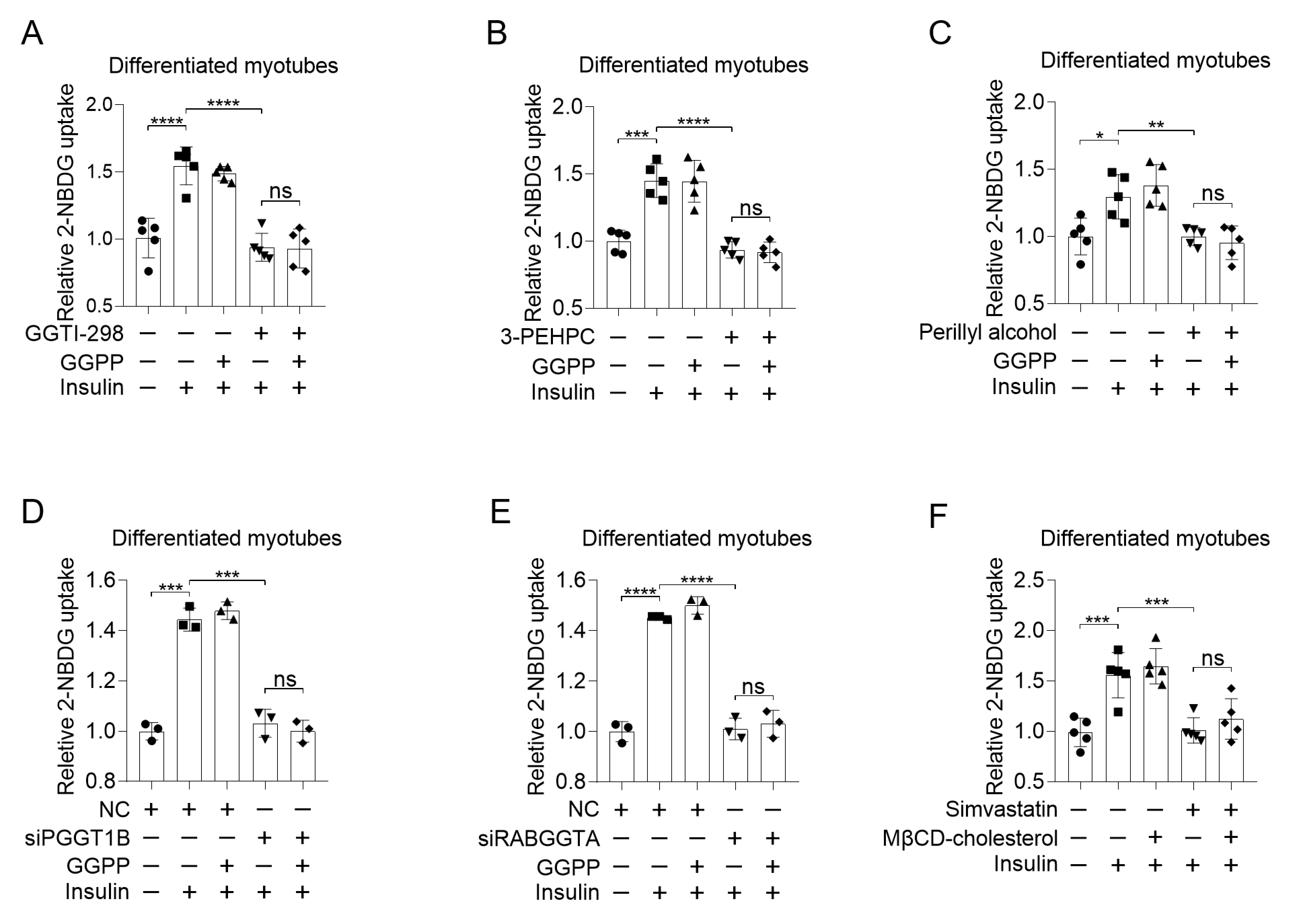

Supplement: Supplementary file 3 — Figure S3 Lipophilic statins suppress insulin sensitivity via inhibiting GGPP production, not cholesterol. (A) C2C12 myotubes were pretreated with 10 μM GGPP, 10 μM GGTI‐298 and 10 μM GGPP combined with 10 μM GGTI‐298 for 24 h, then cells were exposed to 2‐NBDG containing 100 nM insulin for 30 min and 2‐NBDG uptake was measured by fluorescence detection (n = 5). (B) C2C12 myotubes were pretreated with 10 μM GGPP, 1.5 mM 3‐PEHPC and 10 μM GGPP combined with 1.5 mM 3‐PEHPC for 24 h, then cells were exposed to 2‐NBDG containing 100 nM insulin for 30 min and 2‐NBDG uptake was measured by fluorescence detection (n = 5). (C) C2C12 myotubes were pretreated with 10 μM GGPP, 1 mM perillyl alcohol and 10 μM GGPP combined with 1 mM perillyl alcohol for 24 h, then cells were exposed to 2‐NBDG containing 100 nM insulin for 30 min and 2‐NBDG uptake was measured by fluorescence detection (n = 5). (D) C2C12 myotubes were previously transfected with siRNA targeting PGGT1B for 24 h, then cells were treated or not treated with 10 μM GGPP for another 24 h. cells were exposed to 2‐NBDG containing 100 nM insulin for 30 min and 2‐NBDG uptake was measured by fluorescence detection (n = 3). (E) C2C12 myotubes were previously transfected with siRNA targeting RABGGTA for 24 h, then cells were treated or not treated with 10 μM GGPP for another 24 h. cells were exposed to 2‐NBDG containing 100 nM insulin for 30 min and 2‐NBDG uptake was measured by fluorescence detection (n = 3). (F) C2C12 myotubes were pretreated with 10 μM MβCD‐cholesterol, 10 μM simvastatin and 10 μM MβCD‐cholesterol combined with 10 μM simvastatin for 24 h, then cells were exposed to 2‐NBDG containing 100 nM insulin for 30 min and 2‐NBDG uptake was measured by fluorescence detection (n = 5). Data represented the mean ± SEM. Statistical analysis was done with one‐way ANOVA. *P < 0.05; **P < 0.01; ***P < 0.001; ****P < 0.0001. [file JCSM-13-2697-s007.tif]

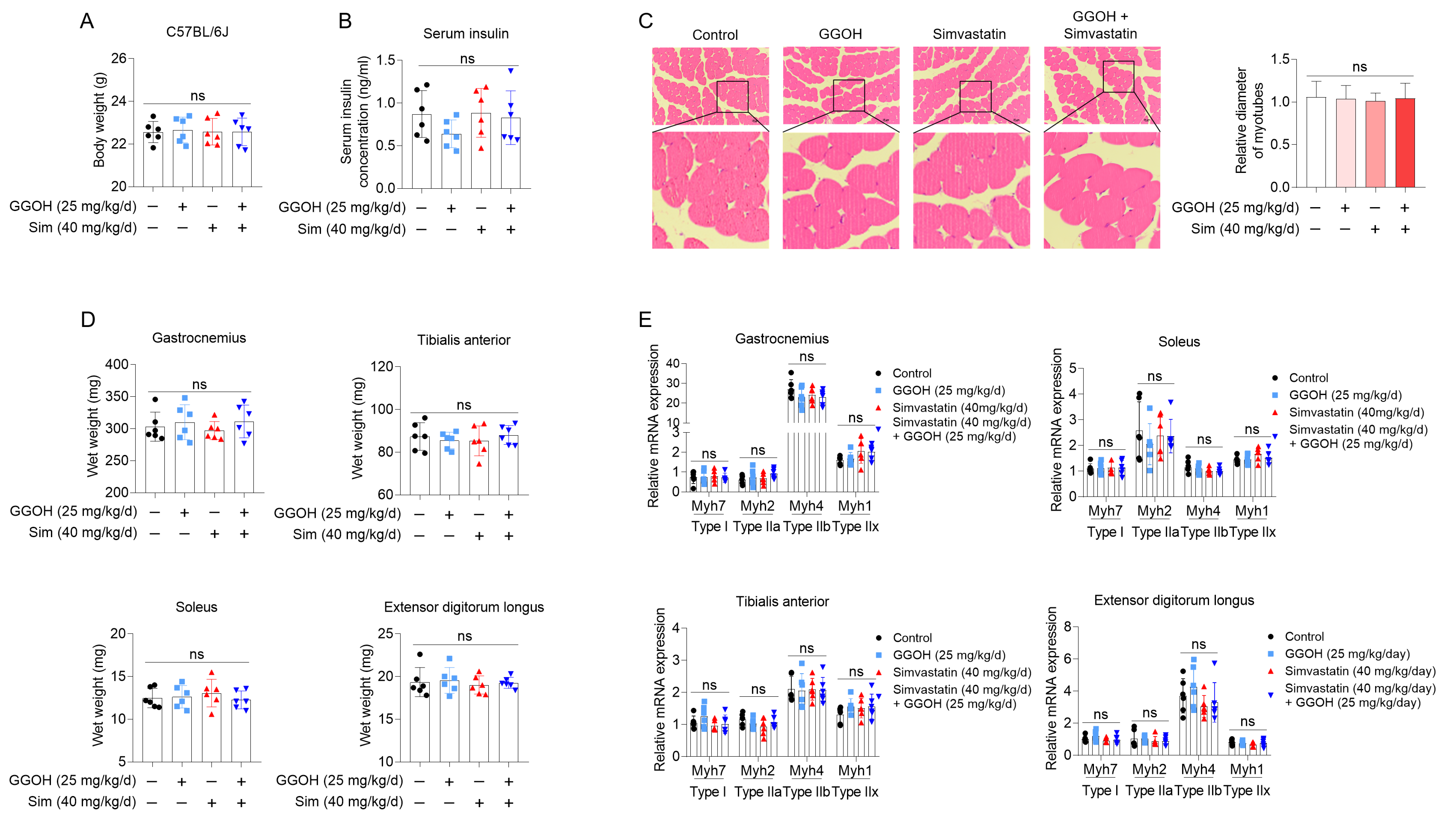

Supplement: Supplementary file 4 — Figure S4 Enhanced insulin sensitivity by GGOH treatment could not be attributed to differences in insulin secretion, muscle mass and muscle fibre type composition. Male C57BL/6J mice (20 ± 2 g) were randomly grouped (n = 6). After administration of GGOH (25 mg/kg/day), simvastatin (40 mg/kg/day), and GGOH combined with simvastatin for 3 weeks, mice were subjected with experiments below. (A) Body weight. (B) Serum insulin levels was measured using ELISA kit. (C) HE staining of the cross section of gastrocnemius. The representative pictures were shown. (D) Wet weight of gastrocnemius, tibialis anterior, soleus and extensor digitus longus. (E) The expression of Myh7, Myh2, Myh4 and Myh1 in gastrocnemius, tibialis anterior, soleus and extensor digitus longus was analyzed by RT‐qPCR. Data represented the mean ± SEM. Statistical analysis was done with one‐way ANOVA. ns meant no significance. [file JCSM-13-2697-s010.tif]

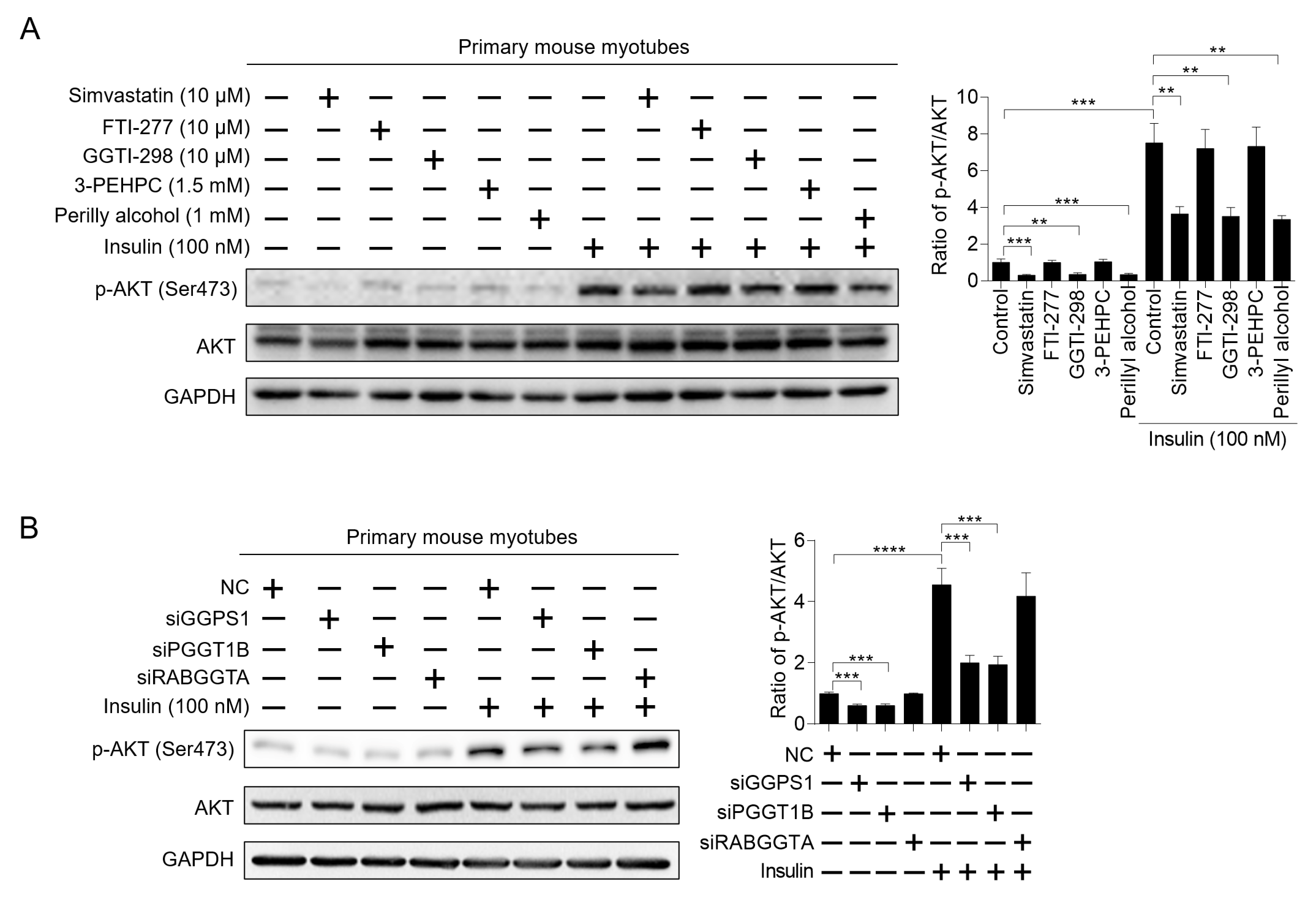

Supplement: Supplementary file 6 — Figure S6 Insulin signaling is not necessary for simvastatin‐caused inhibition of insulin stimulated‐glucose uptake in skeletal muscle cells. (A) Primary mouse myotubes were pretreated with 10 μM simvastatin, 10 μM FTI‐277, 10 μM GGTI‐298, 1.5 mM 3‐PEHPC and 1 mM perillyl alcohol for 24 h. Then cells were incubated with or without 100 nM insulin for 30 min. Total protein was harvested and the expression of indicated proteins was analyzed by western blot, with GAPDH as the loading control (n = 3). (B) Primary mouse myotubes were previously transfected with siRNAs targeting GGPS1, PGGT1B and RABGGTA respectively for 48 h. Then cells were incubated with or without 100 nM insulin for 30 min. Total protein was harvested and the expression of indicated proteins was analyzed by western blot, with GAPDH as the loading control (n = 3). Data represented the mean ± SEM. Statistical analysis was done with one‐way ANOVA. **P < 0.01; ***P < 0.001; ****P < 0.0001; ns meant no significance. [file JCSM-13-2697-s013.tif]

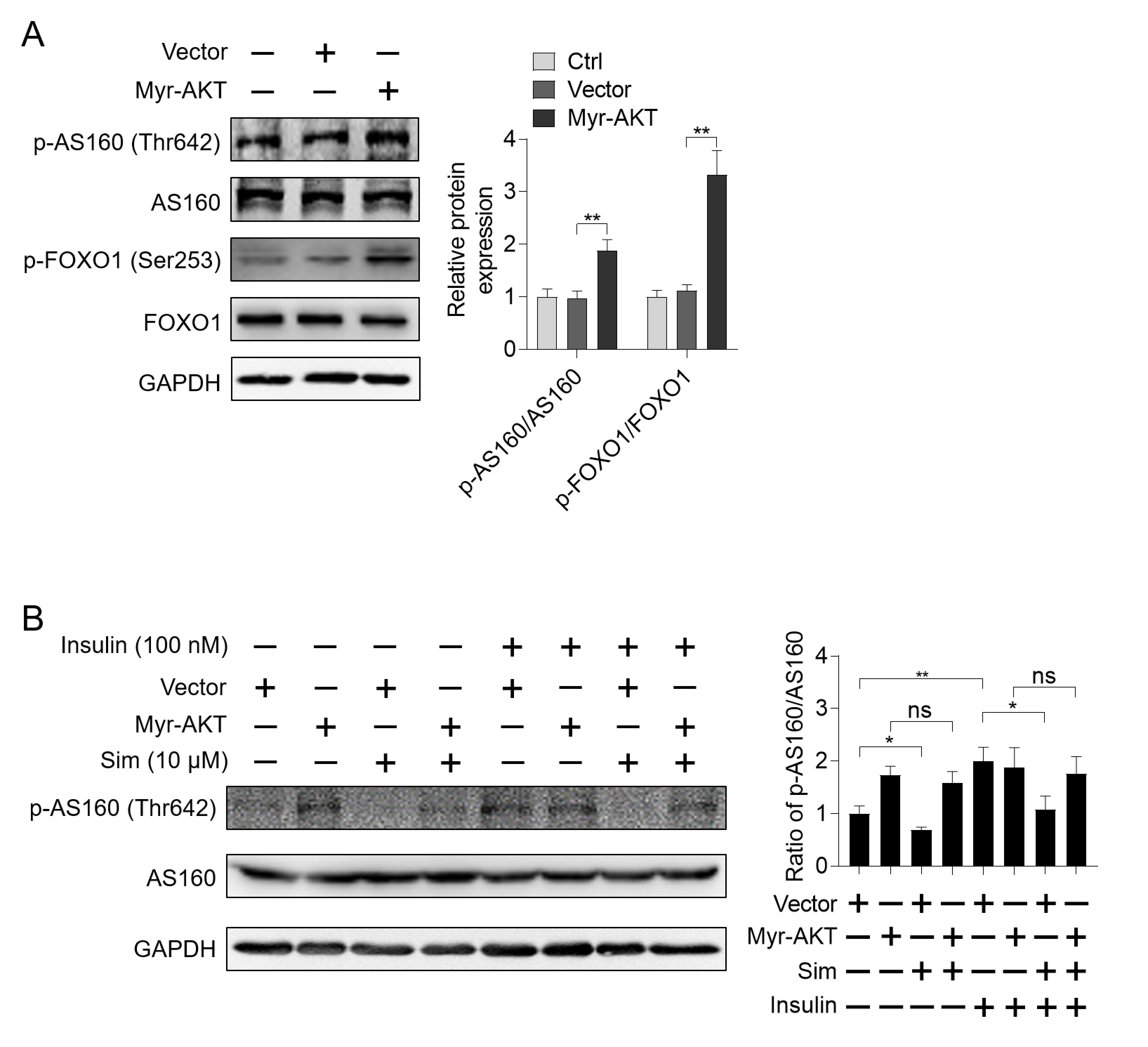

Supplement: Supplementary file 7 — Figure S7 Myr‐AKT reversed the inhibitory effect of simvastatin on insulin signaling. (A) C2C12 myotubes were transfected with 1 μg Myr‐AKT plasmid using Lipofectamine 3000 for 48 h. Protein samples were harvested and the expression of indicated proteins was checked by western blot, with GAPDH as the loading control (n = 3). (B) C2C12 myotubes were previously transfected with or without 1 μg Myr‐AKT plasmid using Lipofectamine 3000 for 48 h, then the cells were treated with or without 10 μM simvastatin for another 24 h. Before the end of the experiment, cells were incubated with or without 100 nM insulin for 30 min, then protein samples were harvested and the expression of indicated proteins was checked by western blot, with GAPDH as the loading control (n = 3). Data represented the mean ± SEM. Statistical analysis was done with one‐way ANOVA. *P < 0.05; **P < 0.01; ns meant no significance. [file JCSM-13-2697-s003.tif]

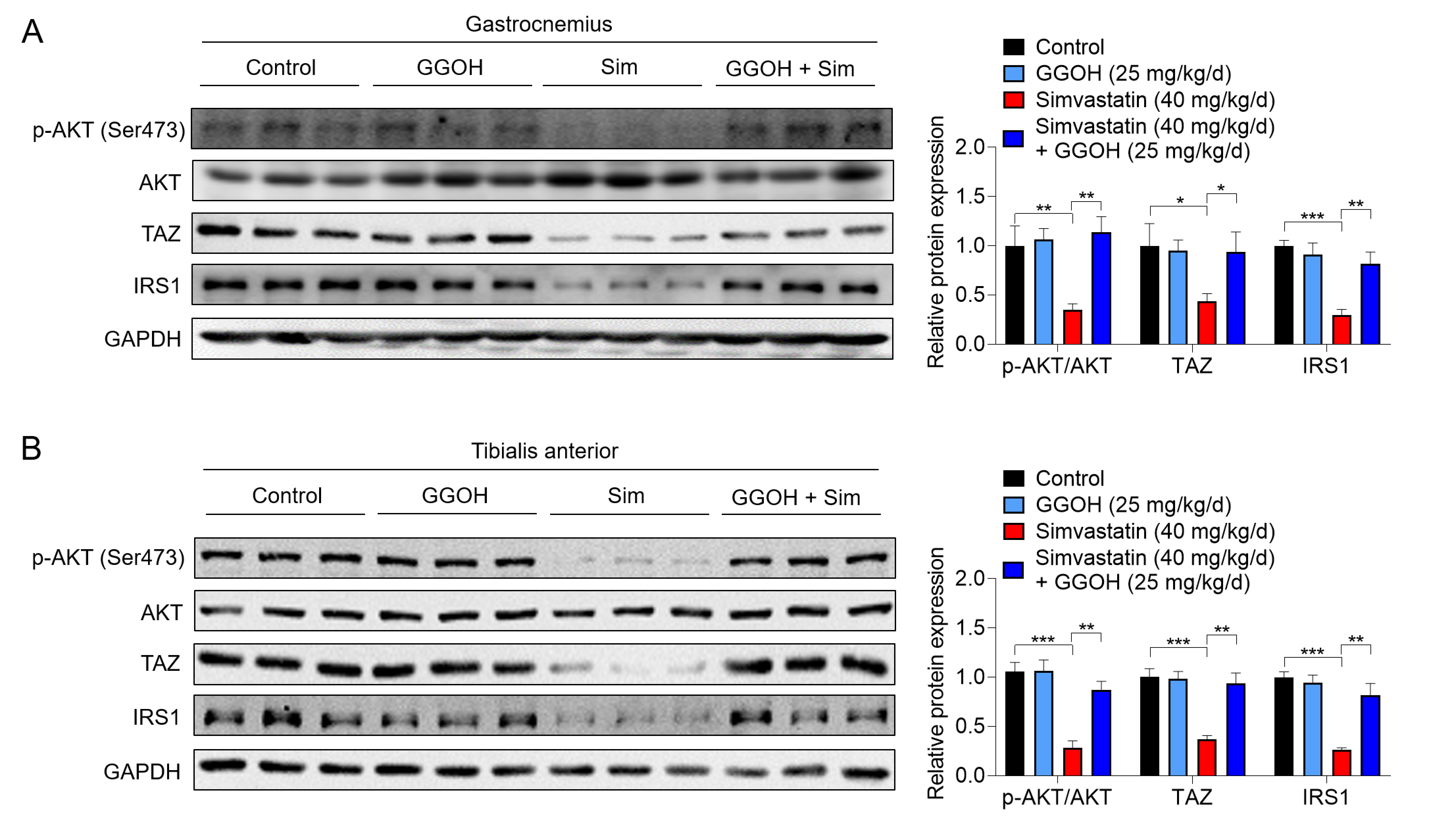

Supplement: Supplementary file 10 — Figure S10 GGOH impeded the inhibition of simvastatin on insulin signaling and TAZ/IRS1 axis in skeletal muscle. (A, B) Male C57BL/6J mice (20 ± 2 g) were randomly grouped (n = 6). After administration of GGOH (25 mg/kg/day), simvastatin (40 mg/kg/day), and GGOH combined with simvastatin for 3 weeks, mice were sacrificed and the expression of indicated proteins in gastrocnemius (A) and tibialis anterior (B) was analyzed by western blot, with GAPDH as the loading control (n = 3). Data represented the mean ± SEM. Statistical analysis was done with one‐way ANOVA. *P < 0.05; **P < 0.01; ***P < 0.001. [file JCSM-13-2697-s017.tif]

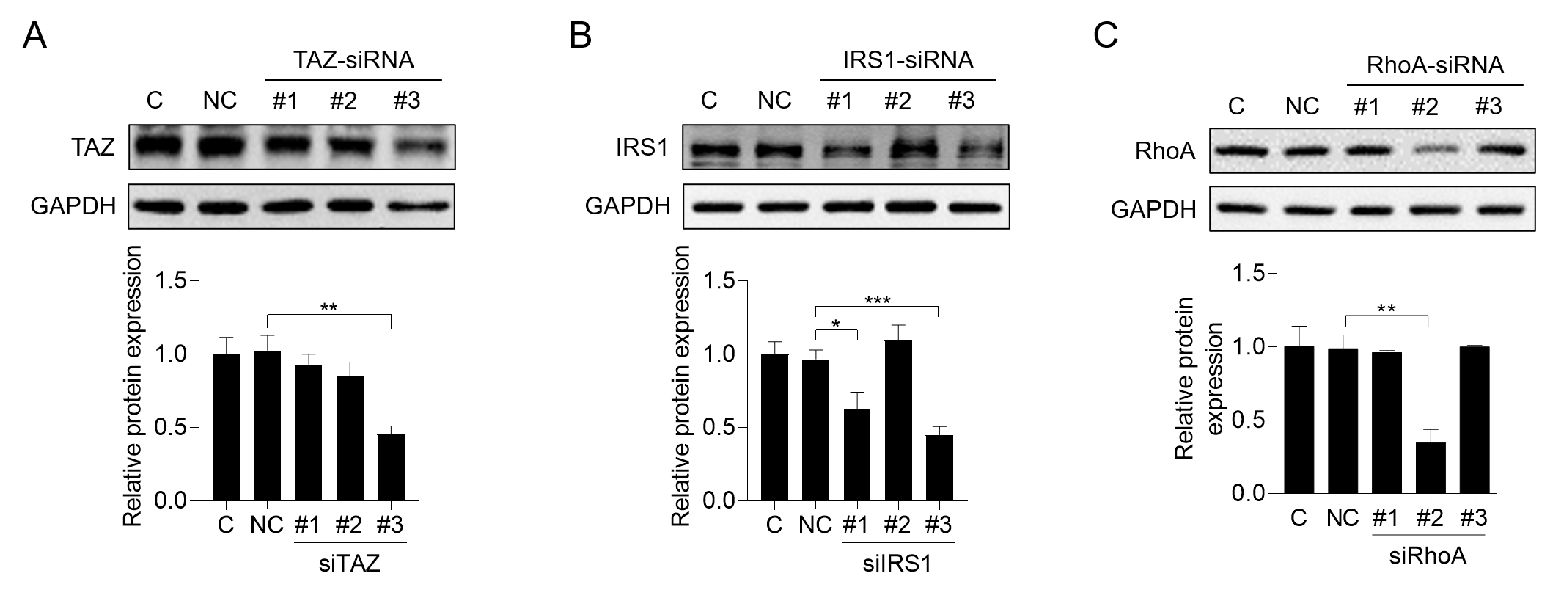

Supplement: Supplementary file 11 — Figure S11 Screening effective siRNA sequences for the knockdown of TAZ, IRS1 and RhoA. C2C12 myoblasts were transfected with siRNAs targeting TAZ (A), IRS1 (B) and RhoA (C) using Lipofectamine 3000 for 48 h. Protein samples were harvested and the knockdown efficiency was checked by western blot, with GAPDH as the loading control (n = 3). Data represented the mean ± SEM. Statistical analysis was done with one‐way ANOVA. *P < 0.05; **P < 0.01; ***P < 0.001. [file JCSM-13-2697-s008.tif]

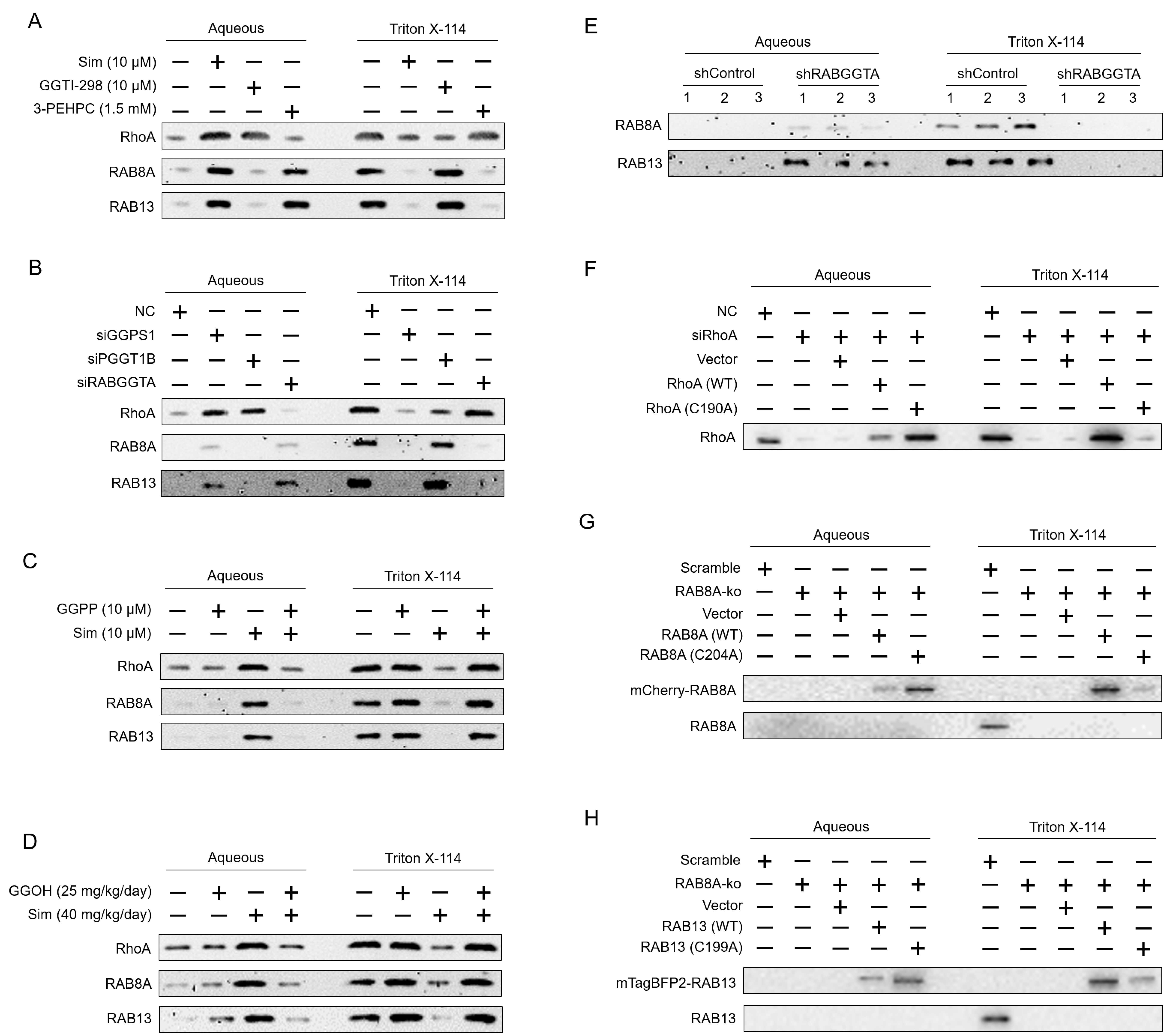

Supplement: Supplementary file 12 — Figure S12 Geranylgeranylation of RhoA, RAB8A and RAB13 was analyzed in C2C12 myotubes and gastrocnemius muscle. (A‐C, F‐H) C2C12 myotubes were treated with 10 μM simvastatin, 10 μM GGTI‐298, 1.5 mM 3‐PEHPC for 24 h (A), C2C12 myotubes were transfected with siRNAs targeting GGPS1, PGGT1B and RABGGTA using Lipofectamine 3000 for 48 h (B), C2C12 myotubes were pretreated with 10 μM GGPP, 10 μM simvastatin and 10 μM GGPP combined with 10 μM simvastatin for 24 h (C), C2C12 myotubes were previously transfected with siRNA targeting RhoA for 24 h, then cells were transfected with vector, RhoA (WT) or RhoA (C190A) plasmids for 48 h (F), RAB8A‐ko C2C12 myotubes were transfected with vector, RAB8A (WT) and RAB8A (C204A) plasmids for 48 h (G), RAB13‐ko C2C12 myotubes were transfected with vector, RAB13 (WT) and RAB13 (C199A) plasmids for 48 h (H). Unprocessed RhoA, RAB8A, RAB13 and geranylgeranylated RhoA, RAB8A, RAB13 in these samples were separated by the Triton X‐114 partition method and analyzed by western blot (n = 3). (D, E) Male C57BL/6J mice (20 ± 2 g) were randomly grouped (n = 6). After administration of GGOH (25 mg/kg/day), simvastatin (40 mg/kg/day), and GGOH combined with simvastatin for 3 weeks. 10 mg gastrocnemius muscle tissue was incised from every mouse. Gastrocnemius muscle tissues from one group were mixed (n = 6) (D). Mice were subjected a week of adjustable feeding, then were divided into two groups including shControl group and shRABGGTA group (n = 6). Posterior limbs of mice in shControl group and shRABGGTA group were infected with control AAV9 and shRABGGTA AAV9 respectively through in situ injection for 4 weeks. 20 mg gastrocnemius muscle tissue was incised from mice (n = 3). Unprocessed RhoA, RAB8A, RAB13 and geranylgeranylated RhoA, RAB8A, RAB13 in gastrocnemius muscle tissues were separated by the Triton X‐114 partition method and analyzed by western blot. [file JCSM-13-2697-s018.tif]

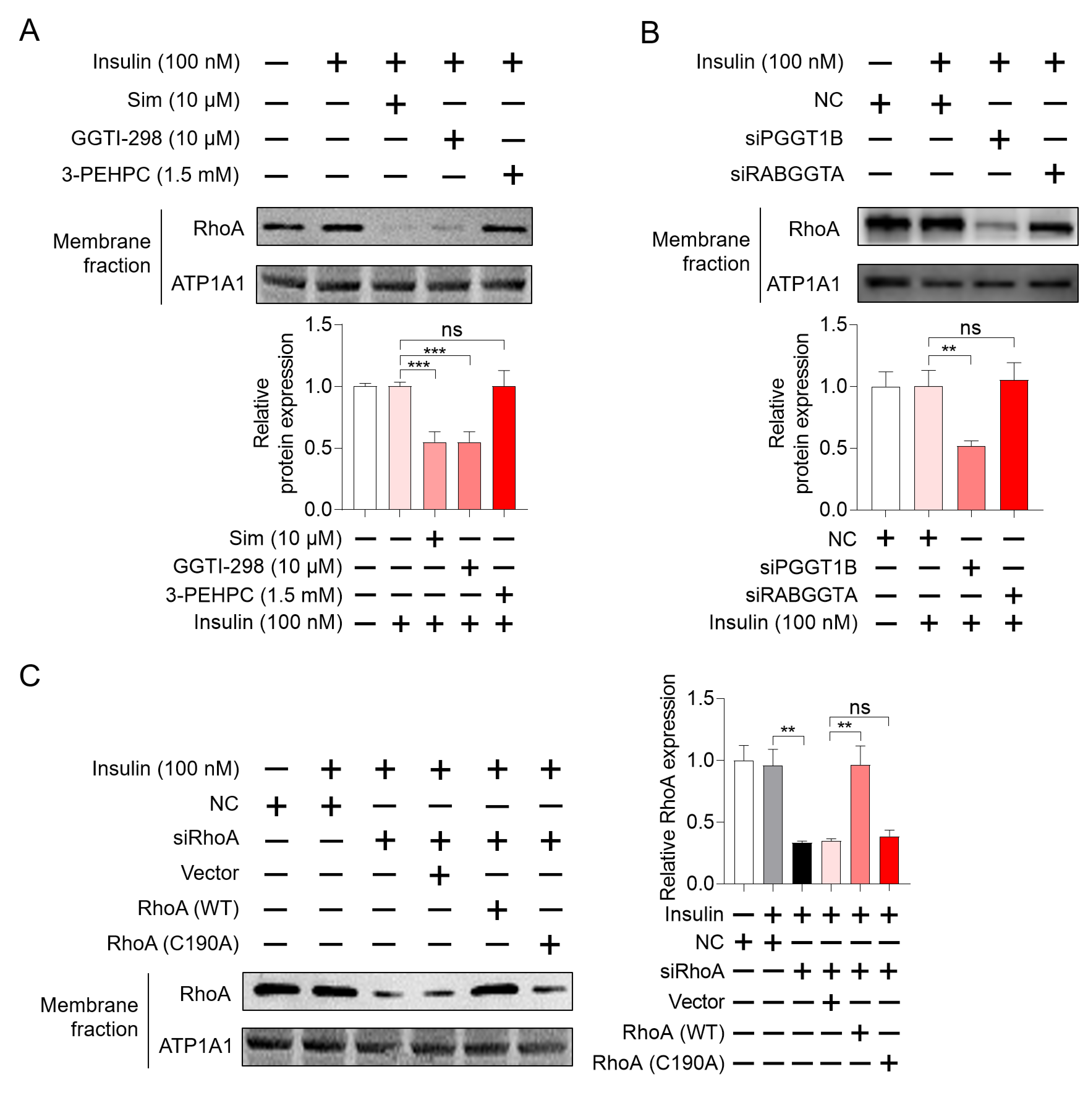

Supplement: Supplementary file 13 — Figure S13 Pharmaceutical and genetic inhibition of GGTase I inhibited the attachment of RhoA to plasma membrane. (A) C2C12 myotubes were treated with 10 μM simvastatin, 10 μM GGTI‐298 and 1.5 mM 3‐PEHPC respectively for 24 h. Before the end of the experiment, cells were incubated with 100 nM insulin for 30 min, then cells were harvested and membrane fractions were extracted. RhoA expression in membrane fraction was analyzed by western blot, with GAPDH as the loading control (n = 3). (B) C2C12 myotubes were transfected with siRNAs targeting PGGT1B and RABGGTA respectively using Lipofectamine 3000 for 48 h. Before the end of the experiment, cells were incubated with 100 nM insulin for 30 min, then cells were harvested and membrane fractions were extracted. RhoA expression in membrane fraction was analyzed by western blot, with GAPDH as the loading control (n = 3). (C) C2C12 myotubes were previously transfected with siRNA targeting RhoA for 24 h, then cells were transfected with vector, RhoA (WT) or RhoA (C190A) plasmids for 48 h. Before the end of the experiment, cells were incubated with 100 nM insulin for 30 min, then plasma membrane protein samples were harvested and the expression of indicated proteins was analyzed by western blot, with GAPDH as the loading control (n = 3). Data represented the mean ± SEM. Statistical analysis was done with one‐way ANOVA. **P < 0.01; ***P < 0.001; ns meant no significance. [file JCSM-13-2697-s012.tif]

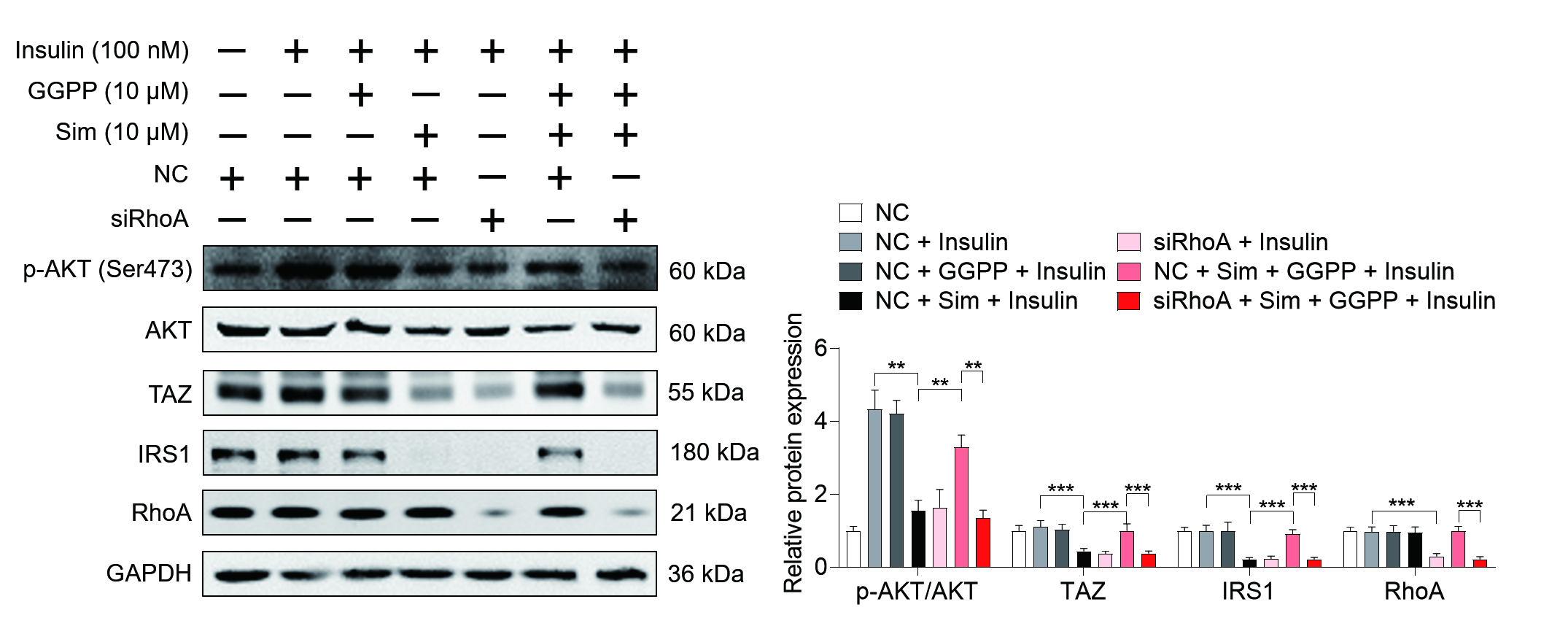

Supplement: Supplementary file 14 — Figure S14 RhoA knockdown attenuated the protective effect of GGPP on simvastatin‐caused inhibition of TAZ/IRS1 axis and insulin signaling. C2C12 myotubes were previously transfected with siRNA targeting RhoA for 48 h, then cells were treated with 10 μM GGPP, 10 μM simvastatin and 10 μM GGPP combined with 10 μM simvastatin respectively for another 24 h. Before the end of the experiment, cells were incubated with 100 nM insulin for 30 min. Protein samples were harvested and the expression of indicated proteins were analyzed by western blot, with GAPDH as the loading control (n = 3). Data represented the mean ± SEM. Statistical analysis was done with one‐way ANOVA. **P < 0.01; ***P < 0.001. [file JCSM-13-2697-s016.tif]

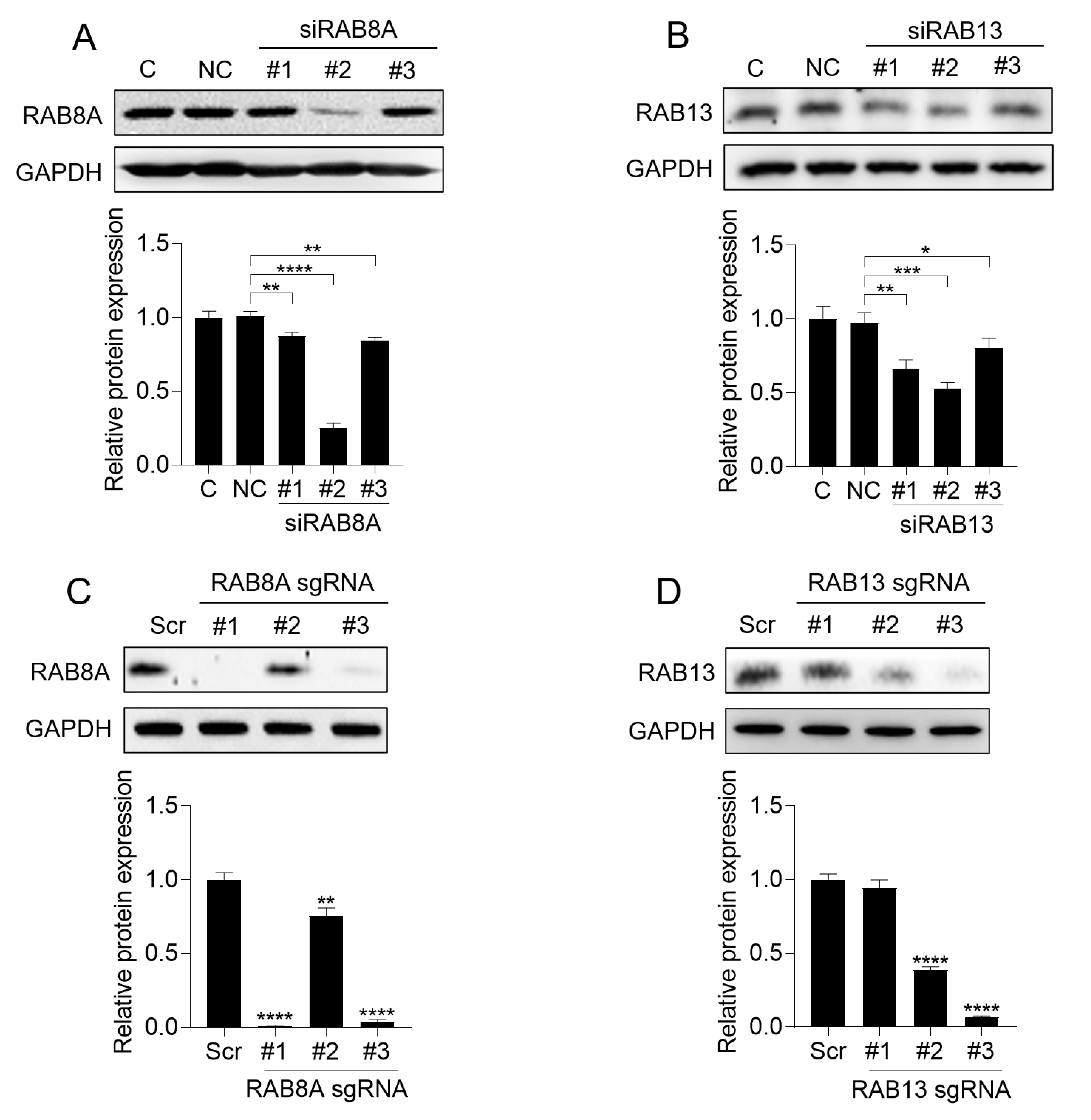

Supplement: Supplementary file 15 — Figure S15 Screening effective siRNA and sgRNA sequences for the knockdown and knockout of RAB8A and RAB13. (A, B) C2C12 myoblasts were transfected with siRNAs targeting RAB8A (A), rab13 (B) using Lipofectamine 3000 for 48 h. Protein samples were harvested and the knockdown efficiency was checked by western blot, with GAPDH as the loading control (n = 3). (C, D) C2C12 myoblasts were previously transfected with lentivirus packaged Cas 9 expressing plasmid DNA for 72 h to establish Cas 9‐expressing C2C12 myoblasts. Then Cas 9‐expressing C2C12 myoblasts were transfected with lentivirus packaged RAB8A sgRNAs (C) or RAB13 sgRNAs (D) for 72 h. Protein samples were harvested and the knockout efficiency was analyzed by western blot, with GAPDH as the loading control (n = 3). Data represented the mean ± SEM. Statistical analysis was done with one‐way ANOVA. *P < 0.05; **P < 0.01; ***P < 0.001; ****P < 0.0001. [file JCSM-13-2697-s005.tif]

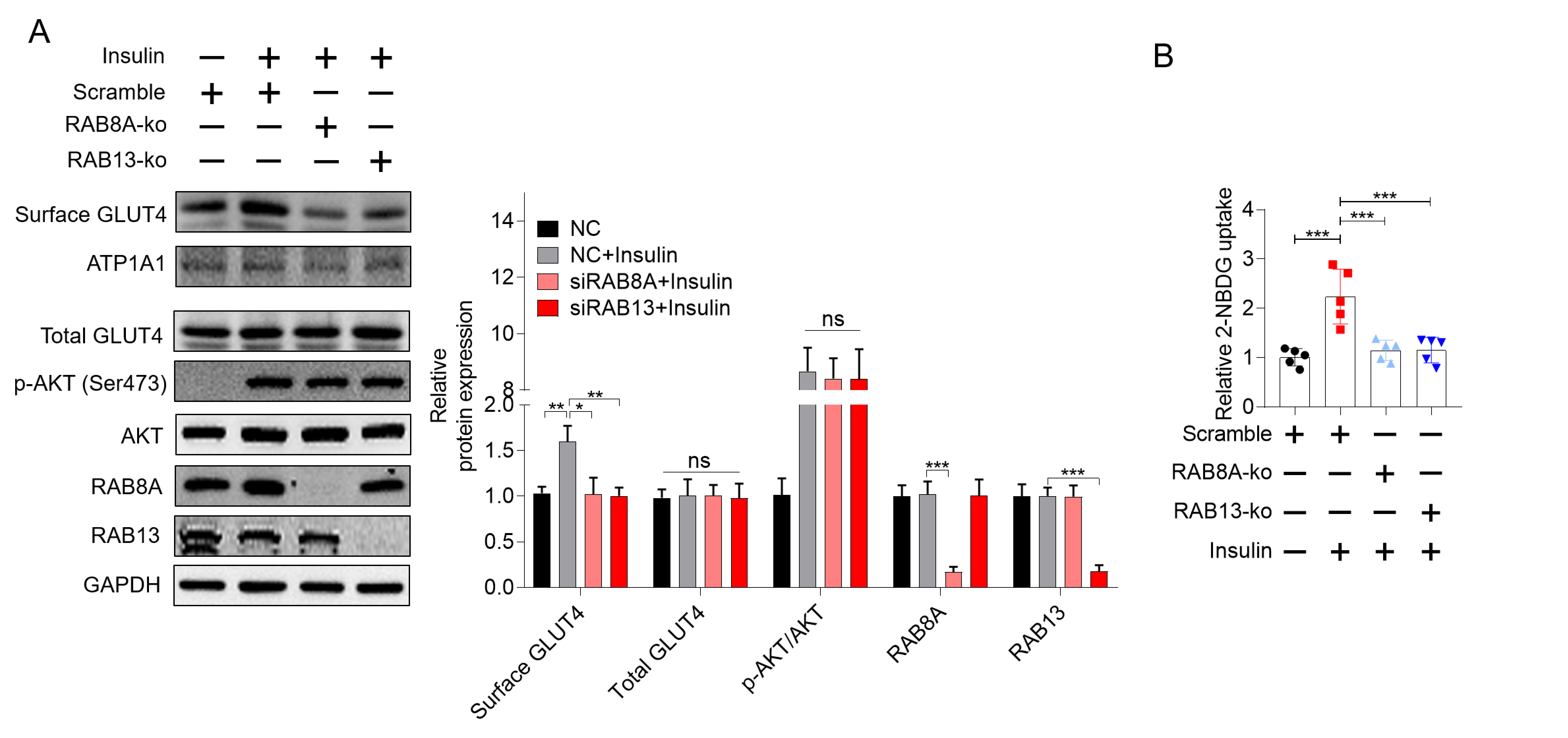

Supplement: Supplementary file 17 — Figure S17 RAB8A knockout and RAB13 knockout suppressed insulin stimulated GLUT4 translocation and concomitant glucose uptake without disturbing insulin signaling. (A) Scramble, RAB8A‐ko and RAB13‐ko C2C12 myotubes were incubated with 100 nM insulin for 30 min. Total protein samples and membrane fraction samples were harvested and the expression of indicated proteins was analyzed by western blot, with GAPDH as the loading control (n = 3). (B) Scramble, RAB8A‐ko and RAB13‐ko C2C12 myotubes were exposed to 2‐NBDG containing 100 nM insulin for 30 min and 2‐NBDG uptake was measured by fluorescence detection (n = 5). Data represented the mean ± SEM. Statistical analysis was done with one‐way ANOVA. **P < 0.01; ***P < 0.001; ****P < 0.0001; ns meant no significance. [file JCSM-13-2697-s014.tif]
