# Supplementary material for: Geranylgeranyl pyrophosphate depletion by statins compromises skeletal muscle insulin sensitivity
Source: J Cachexia Sarcopenia Muscle. 2022 Aug 12;13(6):2697–711. doi: 10.1002/jcsm.13061 (PMC9745480; doi:10.1002/jcsm.13061)
Supplement: Supplementary file 18 — Data S1. Supporting Information [file JCSM-13-2697-s006.docx]

**Supplementary Materials: Supplementary Methods**

**Materials** Simvastatin (S24506), lovastatin (S25584), pravastatin (B27173), rosuvastatin (B70515), atorvastatin (JA791730), fluvastatin (S81136), mevastatin (S26877), pitavastatin (B73103) were purchased from ShanghaiyuanyeBio-TechnologyCo., Ltd (Shanghai, China). GGPP (G6025), GGOH (G3278), insulin (1342106), 2-Deoxy-D-glucose (2-DG, D8375) were purchased from Sigma-Aldrich (California, USA). FTI-277 (HY-15872), GGTI-298 (HY-100876), 3-PEHPC (HY-16011), perillyl alcohol (HY-N7000), 2-Deoxy-2-[(7-nitro-2, 1, 3-benzoxadiazol-4-yl)amino]-D-glucose (2-NBDG, HY-116215), Rhosin hydrochloride (HY-12646) were purchased from MedChemExpress (New Jersey, USA).

**siRNAs, Plasmids, Lentivirus,** **Adeno-associated virus** siRNAs for the silence of mouse GGPS1, PGGT1B, RABGGTA, RAB8A, RAB13 and RhoA were designed and synthesized by Genomeditech (Shanghai, China). siRNAs for the silence of mouse TAZ and IRS1 were designed and synthesized by HANBIO (Shanghai, China). Negative control siRNA was from HANBIO (Shanghai, China). The sequences of siRNAs were shown in Table S1. Constitutively active Myr-AKT in the pcDNA3 expression vector, wild type mouse RhoA in the pcDNA3 expression vector and mouse RhoA (C190A) mutant in the pcDNA3 expression vector were all from HANBIO (Shanghai, China). PGMLV-CMV-MCS-EF1-mCherry-T2A-Puro plasmid DNA encoding wild type mouse RAB8A, PGMLV-CMV-MCS-EF1-mCherry-T2A-Puro plasmid DNA encoding mouse RAB8A (C204A) mutant, PGMLV-CMV-MCS-EF1-ZsGreen1-T2A-Blasticidin plasmid DNA encoding wild type mouse RAB13 and PGMLV-CMV-MCS-EF1-ZsGreen1-T2A-Blasticidin plasmid DNA encoding mouse RAB13 (C199A) mutant were from Genomeditech (Shanghai, China). Lentivirus packaged PGMLV-CMV-M_GLUT4 (P66-Myc-G67)-eGFP-PGK-Puro plasmid DNA was from Genomeditech (Shanghai, China). Adeno-associated virus serotype 9 (AAV9) packaged shRABGGTA plasmid DNA was from HANBIO (Shanghai, China). Cas9-blasticidin lentivirus and lentivirus packaged PGMLV-GM1：U6-gRNA-EF1a-Puro plasmid DNA were from Genomeditech (Shanghai, China). sgRNA target site nucleotide sequences were shown in Table S2.

**Animal Experiments** Male C57BL/6J mice (8 weeks old, 20 ± 2 g) were obtained from Laboratory Animal Center of Yangzhou University (Yangzhou, China). The animal studies were approved by the Animal Ethics Committee of China Pharmaceutical University. All mice were kept in an air-conditioned animal quarter at a temperature of 25 ± 2°C and a relative humidity of 50 ± 10% with 12 h light/dark cycles for 1 week before experiments, and allowed water and standard chow ad libitum.

To determine the diabetogenic effect of simvastatin, 24 male C57BL/6J mice were randomly divided into 2 groups. Simvastatin group (n = 14) was administrated with simvastatin (80 mg/kg/day) by gavage. Control group (n = 10) was administrated with 0.5% CMC-Na by gavage correspondently. Analyses were performed after 8 weeks of administration.

To determine the diabetogenic effect of lovastatin in high fat diet-induced obese mice, 10 male C57BL/6J mice were fed with high fat diet (D12492, Research Diets, New Jersey, USA) for 8 weeks to establish obesity model. Then mice were randomly divided into 2 groups. Lovastatin group (n = 5) was administrated with lovastatin (125 mg/kg/day) by gavage. Control group (n = 5) was administrated with 0.5% CMC-Na by gavage correspondently. Analyses were performed after 8 weeks of administration.

To evaluate the protective effect of geranylgeraniol (GGOH) on simvastatin-induced insulin resistance *in vivo*, 24 male C57BL/6J mice were randomly divided into 4 groups. Control group (n = 6) was administrated with 5% Tween 80 by gavage. GGOH group (n = 6) was administrated with GGOH (25 mg/kg/day) dissolved in 5% Tween 80 by gavage. Simvastatin group (n = 6) was administrated with simvastatin (40 mg/kg/day) dissolved in 5% Tween 80 by gavage. Simvastatin + GGOH group (n = 6) was administrated with simvastatin (40 mg/kg/day) plus GGOH (25 mg/kg/day) by gavage. Analyses were performed after 3 weeks of administration.

To investigate the effect of GGTase II inhibition in skeletal muscle on insulin sensitivity and insulin signaling pathway, adeno-associated virus serotype 9 (AAV9)-mediated knockdown of RABGGTA, the specific α-subunit of GGTase II, was performed. AAV9 infection was addressed *via* multipoint in situ intramuscular injection of two posterior limbs. 12 male C57BL/6J mice were randomly divided into 2 groups. Control group (n = 6) was infected with shControl-AAV9 and RABGGTA knockdown group (n = 6) was infected with shRABGGTA-AAV9. 50 μl AAV9 supernatant for each posterior limb. Analyses were performed after 4 weeks of infection.

**Glucose Tolerance Test** Mice were fasted for 16 h prior to the assay. D-glucose (2 g/kg body weight) was, then, administered intraperitoneally. Mice were bled by cutting their tail tips with a sterilised sharp knife. Blood glucose levels before injection (time 0) and 15, 30, 60, 90 and 120 min after injection were measured using a blood glucose meter (OMRON, Japan). GraphPad Prism 8 software was used to draw the blood glucose levels-time curve and calculate the area under the curve (AUC).

**Insulin Tolerance Test** Mice were fasted for 4 h prior to the assay. Insulin (0.75 U per kg body weight) was, then, administered intraperitoneally. As described for the glucose tolerance test, mouse tail tips were cut with a sterilised sharp knife to induce bleeding, and blood glucose levels were measured using a blood glucose meter at time points as described in glucose tolerance test. GraphPad Prism 8 software was used to draw the blood glucose levels-time curve and calculate the AUC.

**Cell Culture** The mouse myoblast C2C12 cell line was purchased from American Type Culture Collection (ATCC, USA), and the cells were cultured in Dulbecco’s modified Eagle’s medium (DMEM, KeyGEN BioTECH, China) with 10% fetal bovine serum (FBS, Gibco, USA), 1% penicillin-streptomycin. The condition of incubator is 5% CO_2_, 37°C. For differentiating into myotubes, C2C12 myoblasts were switched to the medium with 2% horse serum (Gibco, USA) for 6–10 days.

**Primary culture of mouse myotubes** Primary culture of myotubes was established from satellite cells of 3/6-week-old C57BL/6J mice killed by cervical dislocation. Briefly, muscle tissue was extracted from hindlimbs, was minced in PBS then treated with 1A-type collagenase (1.5 mg/mL, Cat. No C9891, Sigma-Aldrich, USA) and dispase II (2 mg/mL, Sigma-Aldrich, USA) at 37°C for 1 h ± 15 min. The pieces of remaining tissue were triturated with decreasing diameter glass pipettes and several centrifugation and filtration processes were applied to eliminate the last tissue fragments. Myoblast suspension was enriched by a plating step for 45 min at 37°C to decrease the fibroblast fraction. 0.4 × 10^6^ cells were directly seeded on glass-bottom culture dishes previously coated with matrigel (1 mg/mL, Corning, Germany) in a proliferative medium composed of Dulbecco's modified Eagle's medium (DMEM, KeyGEN BioTECH, China) supplemented with 10% FBS (Gibco, USA), 10% horse serum (Gibco, USA) and 1% penicillin/streptomycin (Sigma-Aldrich, USA). Two days after, differentiation was started by replacing proliferative medium by a differentiation medium (DMEM, 2% horse serum, 1% penicillin/streptomycin).

**2-NBDG Uptake Assay** 2-NBDG uptake assay was performed as described earlier([43](#_ENREF_43)) with appropriate changes. Briefly, myotubes were washed with Krebs-Ringer buffer and exposed to 2-NBDG (300 mmol/L) containing 100 nM insulin for 30 min. Then myotubes were rinsed with Krebs buffer, and transferred to a 96 well cell culture plate available for fluorescence detection (Corning, USA). Cultures were excited at 488 nm and the fluorescence was captured at 505–550 nm band-pass filter emission.

***In vivo* 2-DG Uptake Assay** Mice were fasted for 16 hours, then 2-DG (2 g/kg) was intraperitoneally injected. 30 min later, mice were anesthetized and euthanasia. Skeletal muscle including gastrocnemius, tibialis anterior, soleus and extensor digitorum longus were harvested, weighted and stored at -80℃. Glucose Uptake Colorimetric Kit (Biovision, K676-100) was used to analyze glucose uptake according to the manufacturer’s instructions. At the end of the reaction, the absorbance of the samples and standards was measured at 412 nm, using a microplate reader.

**Histological Analysis of Skeletal Muscle** Gastrocnemius muscle were fixed immediately after euthanasia in 4% paraformaldehyde at 4°C overnight and embedded in paraffin wax (Sigma-Aldrich, 327204). Paraffin sections (5 μm) were cut and mounted on glass slides for hematoxylin and eosin (H&E) staining.

**Real-Time Quantitative Polymerase Chain Reaction (RT-qPCR)** Total RNAs were isolated using Trizol reagents (Vazyme, R401-01) and reverse transcribed into cDNA using HiScript Reverse Transcriptase kit (Vazyme, R123-01). Quantitative real-time PCR using SYBR Green Master Mix (Vazyme, Q111-02) was performed with Light-Cycler 480 (Roche Diagnostics GmbH). The primer sequences used in this study were listed in Table S3. The data were analyzed using Microsoft Excel and GraphPad Prism 8.

**Isolation of Plasma Membrane Fractionation** The Plasma Membrane Protein Extraction Kit (ab65400, Abcam) was used to isolate plasma membrane proteins from C2C12 myotubes and primary mouse myotubes according to manufacturer’s instruction.

**Western Blot Analysis** Cells were harvested and suspended in lysis buffer (P0013C, Beyotime Biotechnology) containing protease inhibitor and phosphatase inhibitor. Protein samples were determined using the BCA method (P0009, Beyotime Biotechnology). The cell lysates were denatured at 95°C for 10 min. Proteins were separated by 8-12% SDS-PAGE and transferred to nitrocellulose (NC) membranes (HATF00010, Millipore). The membranes were blocked with 5% nonfat milk (232100, BD Difco TM) in Tris buffered saline (TBS) (A510025, Sangon Biotech) containing 0.075% Tween-20 (93773, Merck) (TBST) for 1 h. The membranes were washed three times with TBST. Then the membrane probing was conducted in antibodies that recognised AKT (AF6261), AS160 (AF7630, Affinity Biosciences, Ohio, USA, 1:1000), GLUT4 (BF1001, Affinity Biosciences, Ohio, USA, 1:1000), GGPS1 (DF12617, Affinity Biosciences, Ohio, USA, 1:1000), RhoA (AF6325, Affinity Biosciences, Ohio, USA, 1:1000), RAB8A (DF9836, Affinity Biosciences, Ohio, USA, 1:1000), RAB13 (DF9813, Affinity Biosciences, Ohio, USA, 1:1000), phospho-AKT (Ser473, 4060, Cell Signaling Technology, Massachusetts, USA, 1:1000), phospho-AS160 (Thr642, 8881, Cell Signaling Technology, Massachusetts, USA, 1:1000), FOXO1 (2880, Cell Signaling Technology, Massachusetts, USA, 1:1000), phospho-FOXO1 (Ser256, 84192, Cell Signaling Technology, Massachusetts, USA, 1:1000), PGGT1B (ab122122, Abcam, Cambridge, UK, 1:1000), RABGGTA (14448-1-AP, ProteinTech Group, Chicago, USA), TAZ (23306-1-AP, ProteinTech Group, Chicago, USA), IRS1 (17509-1-AP, ProteinTech Group, Chicago, USA), GAPDH (10494-1-AP, ProteinTech Group, Chicago, USA), ATP1A1 (14418-1-AP, ProteinTech Group, Chicago, USA) in 4℃ overnight. Next, the membranes were washed three times with TBST and indicated horseradish peroxidase (HRP)-conjugated secondary antibody for 1 h at room temperature. Immunoblots were visualized by chemiluminescence detection kit (180–501, Tanon Science & Technology). Quantitative analysis of each band was performed by Quality One software (BioRad). All the loaded amounts of proteins (total 10–40 μg) were within the linear dynamic range of detection for the Chemiluminescence.

**siRNA and Plasmid Transfection** C2C12 myotubes, C2C12 myoblasts and primary mouse myotubes were transfected with siRNA duplexes or plasmids using Lipofectamine 3000 (Invitrogen, Carlsbad, CA) as described earlier([44](#_ENREF_44)). For transfection of siRNA, 5 nmol of siRNA was used for per well of 6-well cell culture plate. For transfection of plasmids, 1 μg of plasmid DNA was used for per well of 6-well cell culture plate.

**Construction of C2C12 Myoblast Expressing eGFP–GLUT4** The eGFP-GLUT4 lentivirus was purchased from Shanghai GenePharma Co.,Ltd. The cells were seeded at a density of 5 × 10^5^/well in a small culture dish 24 h before transfection to achieve more than 30% confluence. 20 μl eGFP-GLUT4 lentivirus and 20 μl scrambled sequence lentivirus were added into 4 ml fresh medium individually, and then added 4 μl polybrene (Santa Cruz Biotechnology, Santa Cruz, CA) after 24 h-treatment, lentivirus medium was replaced by fresh medium.

**Triton X-114 Partition** Unprocessed RhoA, RAB8A, RAB13 and geranylgeranylated RhoA, RAB8A, RAB13 were separated by the Triton X-114 partition method as described previously with appropriate modification ([45](#_ENREF_45); [46](#_ENREF_46)). Briefly, cells were washed twice with ice-cold PBS and lysed in lysis buffer (50 mM Tris–HCl, pH = 7.4 with 150 mM NaCl, 5 mM MgCl, 1 mM dithiothreitol and 1% Triton X-114) with protease inhibitors for 10 min on ice. Lysates were centrifuged at 20,000 × g at 4°C for 20 min. Protein supernatant was collected and protein concentration was determined by BCA method. Then protein concentration of the supernatant was diluted to 1 mg/ml using lysis buffer. Samples with same volume were subjected with following steps. The supernatant was incubated at 37°C for 2 min, the cloudy state supernatant was centrifuged at 500 × g for 4 min at room temperature. The upper aqueous phase and the lower Triton X-114 phase were collected respectively. The aqueous phase was added with Triton X-114 to a final concentration of 1% and Triton X-114 phase was added with lysis buffer containing no Triton X-114. Both samples were incubated in ice for 5 min until they became clear. Then they were warmed at 37°C, and both phases were collected. Repeat for 3 times. The aqueous phase contained unprocessed RhoA, RAB8A and RAB13 proteins and the Triton X-114 phase contained geranylgeranylated RhoA, RAB8A and RAB13 proteins. Samples with same volume were further subjected to western blot.

**Statistical Analysis** Data were analyzed using the GraphPad Prism 8 software (San Diego, CA, USA). All data were expressed as the means ± standard error of mean (SEM). Student’s *t* test and one-way ANOVA were used to calculate statistical significance. A value of P < 0.05 meant significant; values of P < 0.01, P < 0.001 and P < 0.0001 meant highly significant; ns meant no significance.

**Supplementary Materials: Supplementary Figures**

**Figure S1** Evaluation of the diabetogenic effect of statins *in vivo* and *in vitro.* Statins induced insulin resistance *in vivo* and *in vitro*. Male C57BL/6J mice (20 ± 2 g) were randomly grouped (n = 10 for control group and n = 9 for simvastatin group). Mice in simvastatin group were intragastrically administrated with simvastatin (80 mg/kg/day) for 8 weeks and mice in control group were given 0.5% CMC-Na solution. At the end of the experiment, mice were subjected with experiments as follows. (A) Fasted blood glucose level. (B) GTT and GTT AUC. (C) ITT and ITT AUC. High-fat diet-induced obese male C57BL/6J mice were were randomly grouped (n = 5). Mice in lovastatin group were intragastrically administrated with lovastatin (125 mg/kg/day) for 8 weeks and mice in control group were given 0.5% CMC-Na solution. At the end of the experiment, mice were subjected with experiments as follows. (D) Fasting blood glucose level. (E) GTT and GTT AUC. (F) ITT and ITT AUC. (G) C2C12 myotubes and primary mouse myotubes were treated with 10 μM various statins for 24 h, then cells were exposed to 2-NBDG containing 100 nM insulin for 30 min and 2-NBDG uptake was measured by fluorescence detection (n = 5). (H) C2C12 myotubes were pretreated with simvastatin (2 μM, 10 μM, 50 μM) for 24 h, then cells were exposed to 2-NBDG containing 100 nM insulin for 30 min and 2-NBDG uptake was measured by fluorescence detection (n = 5). Data represented the mean ± SEM. Statistical analysis was done with one-way ANOVA. *P < 0.05; **P < 0.01; ***P < 0.001; ****P < 0.0001.

**Figure S2** Knockdown of GGPS1, PGGT1B and RABGGTA suppressed insulin-stimulated glucose uptake in C2C12 myotubes. C2C12 myoblasts were transfected with siRNAs targeting GGPS1 (A), PGGT1B (B) and RABGGTA (C) using Lipofectamine 3000 for 48 h. Protein samples were harvested and the knockdown efficiency was checked by western blot, with GAPDH as the loading control (n = 3). (D) C2C12 myotubes were previously transfected with siRNAs targeting GGPS1, PGGT1B and RABGGTA respectively for 48 h, then cells were exposed to 2-NBDG containing 100 nM insulin for 30 min and 2-NBDG uptake was measured by fluorescence detection (n = 3). (E) C2C12 myotubes were previously transfected with siRNA targeting GGPS1 for 24 h, then cells were treated or not treated with 10 μM GGPP for another 24 h. Cells were exposed to 2-NBDG containing 100 nM insulin for 30 min and 2-NBDG uptake was measured by fluorescence detection (n = 3). Data represented the mean ± SEM. Statistical analysis was done with one-way ANOVA. **P < 0.01; ****P < 0.0001.

**Figure S3** Lipophilic statins suppress insulin sensitivity *via* inhibiting GGPP production, not cholesterol. (A) C2C12 myotubes were pretreated with 10 μM GGPP, 10 μM GGTI-298 and 10 μM GGPP combined with 10 μM GGTI-298 for 24 h, then cells were exposed to 2-NBDG containing 100 nM insulin for 30 min and 2-NBDG uptake was measured by fluorescence detection (n = 5). (B) C2C12 myotubes were pretreated with 10 μM GGPP, 1.5 mM 3-PEHPC and 10 μM GGPP combined with 1.5 mM 3-PEHPC for 24 h, then cells were exposed to 2-NBDG containing 100 nM insulin for 30 min and 2-NBDG uptake was measured by fluorescence detection (n = 5). (C) C2C12 myotubes were pretreated with 10 μM GGPP, 1 mM perillyl alcohol and 10 μM GGPP combined with 1 mM perillyl alcohol for 24 h, then cells were exposed to 2-NBDG containing 100 nM insulin for 30 min and 2-NBDG uptake was measured by fluorescence detection (n = 5). (D) C2C12 myotubes were previously transfected with siRNA targeting PGGT1B for 24 h, then cells were treated or not treated with 10 μM GGPP for another 24 h. cells were exposed to 2-NBDG containing 100 nM insulin for 30 min and 2-NBDG uptake was measured by fluorescence detection (n = 3). (E) C2C12 myotubes were previously transfected with siRNA targeting RABGGTA for 24 h, then cells were treated or not treated with 10 μM GGPP for another 24 h. cells were exposed to 2-NBDG containing 100 nM insulin for 30 min and 2-NBDG uptake was measured by fluorescence detection (n = 3). (F) C2C12 myotubes were pretreated with 10 μM MβCD-cholesterol, 10 μM simvastatin and 10 μM MβCD-cholesterol combined with 10 μM simvastatin for 24 h, then cells were exposed to 2-NBDG containing 100 nM insulin for 30 min and 2-NBDG uptake was measured by fluorescence detection (n = 5). Data represented the mean ± SEM. Statistical analysis was done with one-way ANOVA. *P < 0.05; **P < 0.01; ***P < 0.001; ****P < 0.0001.

**Figure S4** Enhanced insulin sensitivity by GGOH treatment could not be attributed to differences in insulin secretion, muscle mass and muscle fibre type composition. Male C57BL/6J mice (20 ± 2 g) were randomly grouped (n = 6). After administration of GGOH (25 mg/kg/day), simvastatin (40 mg/kg/day), and GGOH combined with simvastatin for 3 weeks, mice were subjected with experiments below. (A) Body weight. (B) Serum insulin levels was measured using ELISA kit. (C) HE staining of the cross section of gastrocnemius. The representative pictures were shown. (D) Wet weight of gastrocnemius, tibialis anterior, soleus and extensor digitus longus. (E) The expression of Myh7, Myh2, Myh4 and Myh1 in gastrocnemius, tibialis anterior, soleus and extensor digitus longus was analyzed by RT-qPCR. Data represented the mean ± SEM. Statistical analysis was done with one-way ANOVA. ns meant no significance.

**Figure S5** (A) C2C12 myoblasts expressing eGFP-GLUT4 was pretreated with 10 μM simvastatin, 10 μM FTI-277, 10 μM GGTI-298, 1.5 mM 3-PEHPC and 1 mM perillyl alcohol for 24 h. Then cells were incubated with 100 nM insulin for 30 min, localization of GLUT4 was captured by fluorescence confocal microscope (n = 3). (B) C2C12 myoblasts expressing eGFP-GLUT4 were previously transfected with siRNAs targeting GGPS1, PGGT1B and RABGGTA respectively for 48 h, then cells were treated with 100 nM insulin for 30 min, localization of GLUT4 was captured by fluorescence confocal microscope (n = 3). (C) Cells were incubated with 100 nM insulin for 30 min and localization of GLUT4 was captured by fluorescence confocal microscope (n = 3). (D) Cells were incubated with 100 nM insulin for 30 min and localization of GLUT4 was captured by fluorescence confocal microscope (n = 3). The fluorescence intensity was analyzed using ImageJ.

**Figure S6** Insulin signaling is not necessary for simvastatin-caused inhibition of insulin stimulated-glucose uptake in skeletal muscle cells. (A) Primary mouse myotubes were pretreated with 10 μM simvastatin, 10 μM FTI-277, 10 μM GGTI-298, 1.5 mM 3-PEHPC and 1 mM perillyl alcohol for 24 h. Then cells were incubated with or without 100 nM insulin for 30 min. Total protein was harvested and the expression of indicated proteins was analyzed by western blot, with GAPDH as the loading control (n = 3). (B) Primary mouse myotubes were previously transfected with siRNAs targeting GGPS1, PGGT1B and RABGGTA respectively for 48 h. Then cells were incubated with or without 100 nM insulin for 30 min. Total protein was harvested and the expression of indicated proteins was analyzed by western blot, with GAPDH as the loading control (n = 3). Data represented the mean ± SEM. Statistical analysis was done with one-way ANOVA. **P < 0.01; ***P < 0.001; ****P < 0.0001; ns meant no significance.

**Figure S7** Myr-AKT reversed the inhibitory effect of simvastatin on insulin signaling. (A) C2C12 myotubes were transfected with 1 μg Myr-AKT plasmid using Lipofectamine 3000 for 48 h. Protein samples were harvested and the expression of indicated proteins was checked by western blot, with GAPDH as the loading control (n = 3). (B) C2C12 myotubes were previously transfected with or without 1 μg Myr-AKT plasmid using Lipofectamine 3000 for 48 h, then the cells were treated with or without 10 μM simvastatin for another 24 h. Before the end of the experiment, cells were incubated with or without 100 nM insulin for 30 min, then protein samples were harvested and the expression of indicated proteins was checked by western blot, with GAPDH as the loading control (n = 3). Data represented the mean ± SEM. Statistical analysis was done with one-way ANOVA. *P < 0.05; **P < 0.01; ns meant no significance.

**Figure S8** RABGGTA knockdown-induced insulin resistance could not be attributed to differences in insulin secretion, muscle mass and muscle fibre type composition. Mice were subjected a week of adjustable feeding, then were divided into two groups including shControl group and shRABGGTA group (n = 6). Posterior limbs of mice in shControl group and shRABGGTA group were infected with control AAV9 and shRABGGTA AAV9 respectively through in situ injection. 4 weeks after the infection, mice were sacrificed and subjected to a series of analyzes as indicated below. (A) *Invivo* imaging of infected posterior limbs. (B) Body weight. (C) Serum insulin levels. (D) HE staining of the cross section of gastrocnemius. The representative pictures were shown. (E) The wet weights of the gastrocnemius, soleus, tibialis anterior and extensor digitorum longus muscles. (F) Transcript levels of Myh7, Myh2, Myh4 and Myh1 in gastrocnemius, tibialis anterior, soleus and extensor digitorum longus muscles were analyzed by RT-qPCR to determine the composition of muscle fibre types. Data represented the mean ± SEM. Statistical analysis was done with one-way ANOVA. ns meant no significance.

**Figure S9** Simvastatin modulated insulin signaling *via* RhoA geranylgeranylation-mediated TAZ/IRS1 axis in primary mouse myotubes. (A) Primary mouse myotubes were treated with 10 μM simvastatin, 10 μM FTI-277, 10 μM GGTI-298, 1.5 mM 3-PEHPC and 1mM perillyl alcohol for 24 h, protein samples were harvested and the expression of indicated proteins was analyzed by western blot, with GAPDH as the loading control (n = 3). (B) Primary mouse myotubes were transfected with siRNAs specifically targeting GGPS1, PGGT1B and RABGGTA respectively for 48h. Protein samples were harvested and the expression of indicated proteins was analyzed by western blot, with GAPDH as the loading control (n = 3). (C) Primary mouse myotubes was previously transfected with NC or siRNA specifically targeting TAZ and IRS1 respectively for 48 h. Then cells were treated with 10 μM GGPP, 10 μM simvastatin or 10 μM GGPP combined with 10 μM simvastatin for another 24 h. Before the end of the experiment, cells were incubated with 100 nM insulin for 30 min. Then protein samples were harvested and the expression of indicated proteins were analyzed by western blot, with GAPDH as the loading control (n = 3). (D) Primary mouse myotubes were previously transfected with siRNA targeting RhoA for 24 h, then cells were transfected with vector, RhoA (WT) or RhoA (C190A) plasmids for 48 h. Before the end of the experiment, cells were incubated with 100 nM insulin for 30 min, then total protein samples were harvested and the expression of indicated proteins was analyzed by western blot, with GAPDH as the loading control (n = 3). Data represented the mean ± SEM. Statistical analysis was done with one-way ANOVA. *P < 0.05; **P < 0.01; ***P < 0.001; ****P < 0.0001.

**Figure S10** GGOH impeded the inhibition of simvastatin on insulin signaling and TAZ/IRS1 axis in skeletal muscle. *(A, B)* Male C57BL/6J mice (20 ± 2 g) were randomly grouped (n = 6). After administration of GGOH (25 mg/kg/day), simvastatin (40 mg/kg/day), and GGOH combined with simvastatin for 3 weeks, mice were sacrificed and the expression of indicated proteins in gastrocnemius *(A)* and tibialis anterior *(B)* was analyzed by western blot, with GAPDH as the loading control (n = 3). Data represented the mean ± SEM. Statistical analysis was done with one-way ANOVA. *P < 0.05; **P < 0.01; ***P < 0.001.

**Figure S11** Screening effective siRNA sequences for the knockdown of TAZ, IRS1 and RhoA.

C2C12 myoblasts were transfected with siRNAs targeting TAZ (A), IRS1 (B) and RhoA (C) using Lipofectamine 3000 for 48 h. Protein samples were harvested and the knockdown efficiency was checked by western blot, with GAPDH as the loading control (n = 3). Data represented the mean ± SEM. Statistical analysis was done with one-way ANOVA. *P < 0.05; **P < 0.01; ***P < 0.001.

**Figure S12** Geranylgeranylation of RhoA, RAB8A and RAB13 was analyzed in C2C12 myotubes and gastrocnemius muscle. (A-C, F-H) C2C12 myotubes were treated with 10 μM simvastatin, 10 μM GGTI-298, 1.5 mM 3-PEHPC for 24 h (A), C2C12 myotubes were transfected with siRNAs targeting GGPS1, PGGT1B and RABGGTA using Lipofectamine 3000 for 48 h (B), C2C12 myotubes were pretreated with 10 μM GGPP, 10 μM simvastatin and 10 μM GGPP combined with 10 μM simvastatin for 24 h (C), C2C12 myotubes were previously transfected with siRNA targeting RhoA for 24 h, then cells were transfected with vector, RhoA (WT) or RhoA (C190A) plasmids for 48 h (F), RAB8A-ko C2C12 myotubes were transfected with vector, RAB8A (WT) and RAB8A (C204A) plasmids for 48 h (G), RAB13-ko C2C12 myotubes were transfected with vector, RAB13 (WT) and RAB13 (C199A) plasmids for 48 h (H). Unprocessed RhoA, RAB8A, RAB13 and geranylgeranylated RhoA, RAB8A, RAB13 in these samples were separated by the Triton X-114 partition method and analyzed by western blot (n = 3). (D, E) Male C57BL/6J mice (20 ± 2 g) were randomly grouped (n = 6). After administration of GGOH (25 mg/kg/day), simvastatin (40 mg/kg/day), and GGOH combined with simvastatin for 3 weeks. 10 mg gastrocnemius muscle tissue was incised from every mouse. Gastrocnemius muscle tissues from one group were mixed (n = 6) (D). Mice were subjected a week of adjustable feeding, then were divided into two groups including shControl group and shRABGGTA group (n = 6). Posterior limbs of mice in shControl group and shRABGGTA group were infected with control AAV9 and shRABGGTA AAV9 respectively through in situ injection for 4 weeks. 20 mg gastrocnemius muscle tissue was incised from mice (n = 3). Unprocessed RhoA, RAB8A, RAB13 and geranylgeranylated RhoA, RAB8A, RAB13 in gastrocnemius muscle tissues were separated by the Triton X-114 partition method and analyzed by western blot.

**Figure S13** Pharmaceutical and genetic inhibition of GGTase I inhibited the attachment of RhoA to plasma membrane. (A) C2C12 myotubes were treated with 10 μM simvastatin, 10 μM GGTI-298 and 1.5 mM 3-PEHPC respectively for 24 h. Before the end of the experiment, cells were incubated with 100 nM insulin for 30 min, then cells were harvested and membrane fractions were extracted. RhoA expression in membrane fraction was analyzed by western blot, with GAPDH as the loading control (n = 3). (B) C2C12 myotubes were transfected with siRNAs targeting PGGT1B and RABGGTA respectively using Lipofectamine 3000 for 48 h. Before the end of the experiment, cells were incubated with 100 nM insulin for 30 min, then cells were harvested and membrane fractions were extracted. RhoA expression in membrane fraction was analyzed by western blot, with GAPDH as the loading control (n = 3). (C) C2C12 myotubes were previously transfected with siRNA targeting RhoA for 24 h, then cells were transfected with vector, RhoA (WT) or RhoA (C190A) plasmids for 48 h. Before the end of the experiment, cells were incubated with 100 nM insulin for 30 min, then plasma membrane protein samples were harvested and the expression of indicated proteins was analyzed by western blot, with GAPDH as the loading control (n = 3). Data represented the mean ± SEM. Statistical analysis was done with one-way ANOVA. **P < 0.01; ***P < 0.001; ns meant no significance.

**Figure S14** RhoA knockdown attenuated the protective effect of GGPP on simvastatin-caused inhibition of TAZ/IRS1 axis and insulin signaling. C2C12 myotubes were previously transfected with siRNA targeting RhoA for 48 h, then cells were treated with 10 μM GGPP, 10 μM simvastatin and 10 μM GGPP combined with 10 μM simvastatin respectively for another 24 h. Before the end of the experiment, cells were incubated with 100 nM insulin for 30 min. Protein samples were harvested and the expression of indicated proteins were analyzed by western blot, with GAPDH as the loading control (n = 3). Data represented the mean ± SEM. Statistical analysis was done with one-way ANOVA. **P < 0.01; ***P < 0.001.

**Figure S15** Screening effective siRNA and sgRNA sequences for the knockdown and knockout of RAB8A and RAB13. (A, B) C2C12 myoblasts were transfected with siRNAs targeting RAB8A (A), rab13 (B) using Lipofectamine 3000 for 48 h. Protein samples were harvested and the knockdown efficiency was checked by western blot, with GAPDH as the loading control (n = 3). (C, D) C2C12 myoblasts were previously transfected with lentivirus packaged Cas 9 expressing plasmid DNA for 72 h to establish Cas 9-expressing C2C12 myoblasts. Then Cas 9-expressing C2C12 myoblasts were transfected with lentivirus packaged RAB8A sgRNAs (C) or RAB13 sgRNAs (D) for 72 h. Protein samples were harvested and the knockout efficiency was analyzed by western blot, with GAPDH as the loading control (n = 3). Data represented the mean ± SEM. Statistical analysis was done with one-way ANOVA. *P < 0.05; **P < 0.01; ***P < 0.001; ****P < 0.0001.

**Figure S16** Geranylgeranylation of RAB8A was critical for insulin-stimulated GLUT4 translocation and concomitant glucose uptake in skeletal muscle cells. *(A)* Primary mouse myotubes were transfected with siRNAs specifically targeting RAB8A and RAB13 respectively for 48 h. Then cells were incubated with 100 nM insulin for 30 min. Total protein samples and plasma membrane fraction samples were harvested and the expression of indicated proteins were analyzed by western blot, with GAPDH as the loading control (n = 3). *(B)* Primary mouse myotubes were transfected with siRNAs specifically targeting RAB8A and RAB13 respectively for 48 h. Then cells were exposed to 2-NBDG containing 100 nM insulin for 30 min and 2-NBDG uptake was measured by fluorescence detection (n = 3). *(C)* Primary mouse myotubes were previously transfected with RAB8A siRNA for 24 h for RAB8A knockdown. Then these cells were transfected with vector, RAB8A (WT) and RAB8A (C204A) plasmids for another 48 h. Next, cells were incubated with 100 nM insulin for 30 min. Total protein samples and plasma membrane fraction samples were harvested and the expression of indicated proteins were analyzed by western blot, with GAPDH as the loading control (n = 3). *(D)* Primary mouse myotubes were previously transfected with RAB8A siRNA for 24 h for RAB8A knockdown. Then these cells were transfected were transfected with vector, RAB8A (WT) and RAB8A (C204A) plasmids for 48 h. Next, cells were exposed to 2-NBDG containing 100 nM insulin for 30 min and 2-NBDG uptake was measured by fluorescence detection (n = 3). *(E)* Primary mouse myotubes were previously transfected with RAB13 siRNA for 24 h for RAB8A knockdown. Then these cells were transfected with vector, RAB13 (WT) and RAB13 (C199A) plasmids for another 48 h. Next, cells were incubated with 100 nM insulin for 30 min. Total protein samples and plasma membrane fraction samples were harvested and the expression of indicated proteins were analyzed by western blot, with GAPDH as the loading control (n = 3). *(F)* Primary mouse myotubes were previously transfected with RAB8A siRNA for 24 h for RAB13 knockdown. Then these cells were transfected were transfected with vector, RAB13 (WT) and RAB13 (C199A) plasmids for 48 h. Next, cells were exposed to 2-NBDG containing 100 nM insulin for 30 min and 2-NBDG uptake was measured by fluorescence detection (n = 3). Data represented the mean ± SEM. Statistical analysis was done with one-way ANOVA. *P < 0.05; **P < 0.01; ***P < 0.001; ****P < 0.0001; ns meant no significance.

**Figure S17** RAB8A knockout and RAB13 knockout suppressed insulin stimulated GLUT4 translocation and concomitant glucose uptake without disturbing insulin signaling. (A) Scramble, RAB8A-ko and RAB13-ko C2C12 myotubes were incubated with 100 nM insulin for 30 min. Total protein samples and membrane fraction samples were harvested and the expression of indicated proteins was analyzed by western blot, with GAPDH as the loading control (n = 3). (B) Scramble, RAB8A-ko and RAB13-ko C2C12 myotubes were exposed to 2-NBDG containing 100 nM insulin for 30 min and 2-NBDG uptake was measured by fluorescence detection (n = 5). Data represented the mean ± SEM. Statistical analysis was done with one-way ANOVA. **P < 0.01; ***P < 0.001; ****P < 0.0001; ns meant no significance.

**Supplementary Materials: Supplementary Tables**

Table S1. Oligo sequences for gene silence

| Specifies | Gene name | siRNA sequences (5′ to 3′) |
| --- | --- | --- |
| Mus musculus | NC | Sense: UUCUCCGAACGUGUCACGU dTdT  Antisense: ACGUGACACGUUCGGAGAA dTdT |
|  | GGPS1-si-1  GGPS1-si-2  GGPS1-si-3 | Sense: GCAAACUUUCACAGGCAUUUA tt  Antisense: UAAAUGCCUGUGAAAGUUUGC tt  Sense: GCAGCUGUUCUCUGAUUACAA tt  Antisense: UUGUAAUCAGAGAACAGCUGC tt  Sense: GCCAGUUUACUCAUUGAUGA tt  Antisense: AUCAUCAAUGAGUAAACUGG tt |
|  | PGGT1B-si-1  PGGT1B-si-2  PGGT1B-si-3 | Sense: UGGUGAACAAAGACGAUAUAA tt  Antisense: UUAUAUCGUCUUUGUUCACCA tt  Sense: GACUAUAUGUUUGUAGGUUAU tt  Antisense: AUAACCUACAAACAUAUAGUC tt  Sense: CACUGUGCCUGAUGGGUAAAC tt  Antisense: GUUUACCCAUCAGGCACAGUG tt |
|  | RABGGTA-si-1  RABGGTA-si-2  RABGGTA-si-3 | Sense: UCCGAGCUUGAGUCCUGUAAG tt  Antisense: CUUACAGGACUCAAGCUCGGA tt  Sense: GCUGGAUGAAUCCGUCCUA tt  Antisense: UAGGACGGAUUCAUCCAGC tt  Sense: GCGUUUGGCUGAGAUGCUA tt  Antisense: UAGCAUCUCAGCCAAACGC tt |
|  | RAB8A-si-1  RAB8A-si-2  RAB8A-si-3 | Sense: CGGAAUUGGAUUCGGAACAUU tt  Antisense: AAUGUUCCGAAUCCAAUUCCG tt  Sense: CUCGAUGGCAAGAGGAUUAAA tt  Antisense: UUUAAUCCUCUUGCCAUCGAG tt  Sense: CGCCUUCAACUCCACAUUCAU tt  Antisense: AUGAAUGUGGAGUUGAAGGCG tt |
|  | RAB13-si-1  RAB13-si-2  RAB13-si-3 | Sense: AGAUCCGAACCGUGGACAUAG tt  Antisense: CUAUGUCCACGGUUCGGAUCU tt  Sense: CGAGAAUAUUCAGAACUGGAU tt  Antisense: AUCCAGUUCUGAAUAUUCUCG tt  Sense: GAGCAUUUCUUGCCUCCUAUU tt  Antisense: AAUAGGAGGCAAGAAAUGCUC tt |
|  | TAZ-si-1  TAZ-si-2  TAZ-si-3 | Sense: CCUUCUUUAAGGAGCCCGA dTdT  Antisense: UCGGGCUCCUUAAAGAAGG dTdT  Sense: CAGCCGAAUCUCGCAAUGA dTdT  Antisense: UCAUUGCGAGAUUCGGCUG dTdT  Sense: GUGAUGAAUCAGCCUCUGA dTdT  Antisense: UCAGAGGCUGAUUCAUCAC dTdT |
|  | IRS1-si-1  IRS1-si-2  IRS1-si-3 | Sense: CGAGACGAACACUUUGCCA dTdT  Antisense: UGGCAAAGUGUUCGUCUCG dTdT  Sense: CCCAGGAGAAUAUGUGAAU dTdT  Antisense: AUUCACAUAUUCUCCUGGG dTdT  Sense: CGGUCCUCUCUUACUACUC dTdT  Antisense: GAGUAGUAAGAGAGGACCG dTdT |
|  | RhoA-si-1  RhoA-si-2  RhoA-si-3 | Sense: GACAUGCUUGCUCAUAGUC tt  Antisense: GACUAUGAGCAAGCAUGUC tt  Sense: AUUGACAGCCCUGAUAGUUUA tt  Antisense: UAAACUAUCAGGGCUGUCAAU tt  Sense: GUCAAGCAUUUCUGUCCAAAU tt  Antisense: AUUUGGACAGAAAUGCUUGAC tt |

Table S2. sgRNA target site nucleotide sequences

| Gene name | gRNA sequence (5′ to 3′) | PAM |
| --- | --- | --- |
| Scramble | ACGGAGGCTAAGCGTCGCAA |  |
| Mouse_RAB8A sgRNA1 | ATTAGGACCATAGAGCTCGA | TGG |
| Mouse_RAB8A sgRNA2 | CCATAGAGCTCGATGGCAAG | AGG |
| Mouse_RAB8A sgRNA3 | CCTCTTGCCATCGAGCTCTA | TGG |
| Mouse_RAB13 sgRNA1 | TGACATGGAGGCCAAGCGGC | AGG |
| Mouse_RAB13 sgRNA2 | GAGGCGCTCCACTCCCGCAG | AGG |
| Mouse_RAB13 sgRNA3 | GGAGTGGAGCGCCTCCTGCT | GGG |

Table S3. Primer sequences for RT-qPCR

| Specifies | Gene name | Sequence of forward and reverse primers (5′ to 3′) |
| --- | --- | --- |
| Mus musculus | Myh1 | Forward Sequence: AGAGCCAAGAGGAAACTGGAGG  Reverse Sequence: CTCGTCCTCAATCTTGCTCTGC |
|  | Myh2 | Forward Sequence: GCGACTTGAAGTTAGCCCAGGA  Reverse Sequence: CTCGTCCTCAATCTTGCTCTGC |
|  | Myh4 | Forward Sequence: AGAGCCAAGAGGAAACTGGAGG  Reverse Sequence: CTCGTCCTCAATCTTGCTCTGC |
|  | Myh7 | Forward Sequence: GCTGGAAGATGAGTGCTCAGAG  Reverse Sequence: TCCAAACCAGCCATCTCCTCTG |
|  | GAPDH | Forward Sequence: CATCACTGCCACCCAGAAGACTG  Reverse Sequence: ATGCCAGTGAGCTTCCCGTTCAG |
